# ShuTu: Open-Source Software for Efficient and Accurate Reconstruction of Dendritic Morphology

**DOI:** 10.3389/fninf.2019.00068

**Published:** 2019-10-31

**Authors:** Dezhe Z. Jin, Ting Zhao, David L. Hunt, Rachel P. Tillage, Ching-Lung Hsu, Nelson Spruston

**Affiliations:** ^1^Department of Physics and Center for Neural Engineering, The Pennsylvania State University, University Park, PA, United States; ^2^Janelia Research Campus, Howard Hughes Medical Institute, Ashburn, VA, United States

**Keywords:** neuron morphology, reconstruction, dendrite, automatic reconstruction method, software

## Abstract

Neurons perform computations by integrating inputs from thousands of synapses—mostly in the dendritic tree—to drive action potential firing in the axon. One fruitful approach to studying this process is to record from neurons using patch-clamp electrodes, fill the recorded neurons with a substance that allows subsequent staining, reconstruct the three-dimensional architectures of the dendrites, and use the resulting functional and structural data to develop computer models of dendritic integration. Accurately producing quantitative reconstructions of dendrites is typically a tedious process taking many hours of manual inspection and measurement. Here we present ShuTu, a new software package that facilitates accurate and efficient reconstruction of dendrites imaged using bright-field microscopy. The program operates in two steps: (1) automated identification of dendritic processes, and (2) manual correction of errors in the automated reconstruction. This approach allows neurons with complex dendritic morphologies to be reconstructed rapidly and efficiently, thus facilitating the use of computer models to study dendritic structure-function relationships and the computations performed by single neurons.

## Introduction

The geometry of dendritic arbors directly influences synaptic integration and the resultant firing patterns of neurons (Henze et al., 1996; Mainen and Sejnowski, 1996; Stuart and Spruston, 1998; Krichmar et al., 2002). Dendritic morphologies vary widely across and within regions of the brain (Parekh and Ascoli, 2013), so consideration of morphology is an important aspect of understanding the mechanisms by which different neurons carry out their unique functions. Intracellular recording of neurons is a common technique for studying dendritic integration of input signals (Hamill et al., 1981; Stuart and Spruston, 1998). To fully understand the implications of these experiments, numerical simulations of the recorded neurons are often needed (Jaeger, 2001; Krichmar et al., 2002; Gidon and Segev, 2012; Menon et al., 2013). Informative simulations require accurate reconstructions of the geometry of the recorded neurons, including branching structures and diameters of the branches.

The traditional method of reconstructing neuron morphology requires intensive human labor (Zandt et al., 2017). A slide containing a neuron filled with biocytin is mounted on a motorized stage and imaged using a video camera mounted to a bright-field microscope. The neuron image is displayed on a computer screen, and the reconstruction is done manually. The user clicks the mouse along the images of dendritic branches on the screen. While clicking, the user adjusts the cursor size to match the diameters, and turns the focus knob (*z* position) on the microscope to keep the branches in focus. Each click records the *x*, *y*, and *z* positions and the radius *r* at a single point, and connects the point to the previously clicked point. Bifurcations are marked and followed up sequentially. The morphology is recorded in a series of these clicked points.

Manual reconstruction in this way is computationally straightforward. Since it requires no image storage or processing, the computational demand is minimal. However, there are several drawbacks, especially when the accuracy of reconstruction is crucial. Repetitive clicking while measuring the radii and turning the focus knob makes manual reconstruction labor-intensive and time-consuming. The problem is exacerbated at high magnification. To see fine processes of neurons, it is desirable to image neurons with an objective at 100× magnification and a large numerical aperture (Jaeger, 2001; Brown et al., 2011). In our experience, however, it can take 10–15 h or more of continuous work to reconstruct the dendritic tree of a pyramidal neuron in this way. Over this period of time, instability of the sample in the microscope can lead to problems. Furthermore, the accuracy of the reconstruction can suffer from fatigue-induced mistakes. Another problem with manual reconstruction is that the accuracy is hard to check independently because it is difficult to precisely align the previous reconstruction with the neuron image after remounting the slide.

Automatic reconstruction of neuron morphology using computer algorithms promises to reduce manual labor and increase productivity. There have been intensive efforts toward this goal for decades (Capowski, 1983). Recent work includes open-source projects, such as the Digital Reconstruction of Axonal and Dendritic Morphology Challenge (DIADEM) (Gillette et al., 2011a,b; Liu, 2011; Svoboda, 2011) and the BigNeuron project (Peng et al., 2015). Commercial software is also moving in this direction. A recent paper reviews many algorithms for automatic reconstruction proposed over the years (Acciai et al., 2016). In our experience, however, available software still suffers from a variety of problems, including limited automation and tedious approaches for error correction. In particular, algorithms for automatic reconstruction of neurons stained with a dark reaction product are lacking. Thus, we sought to develop an open-source software platform that would overcome these limitations. In this paper, we describe our open-source software package, ShuTu (Chinese for “dendrite”)—a system for reconstructing biocytin-filled neurons efficiently and accurately by combining a novel automatic tracing algorithm and a graphic user interface (GUI) designed for efficient manual editing and error corrections. To avoid the impression of marketing our software, we make no attempt to compare it to other open-source or commercial software; instead, we encourage others to try it and judge for themselves.

## Results

We demonstrate the use and design of ShuTu by going through the steps involved in reconstructing a single CA3 pyramidal neuron from a mouse hippocampal slice. We then present reconstruction results for other cell types as well. Neurons were stained following patch-clamp recordings in brain slices prepared from 17 to 30 days-old male mice (C57Bl/6), using biocytin-containing intracellular solution and stained with a dark reaction product. Following recording and staining, neuron reconstruction proceeded according to the following steps: (1) image acquisition; (2) image processing; (3) automated reconstruction; (4) manual editing and error correction. Additional details regarding slice recordings and computer systems requirements are provided in the Materials and Methods section. Operational commands for ShuTu are provided in Appendix 1. Technical details regarding the algorithms used in ShuTu for automated reconstruction are provided in Appendix 2.

### Image Acquisition

**Figure 1:**
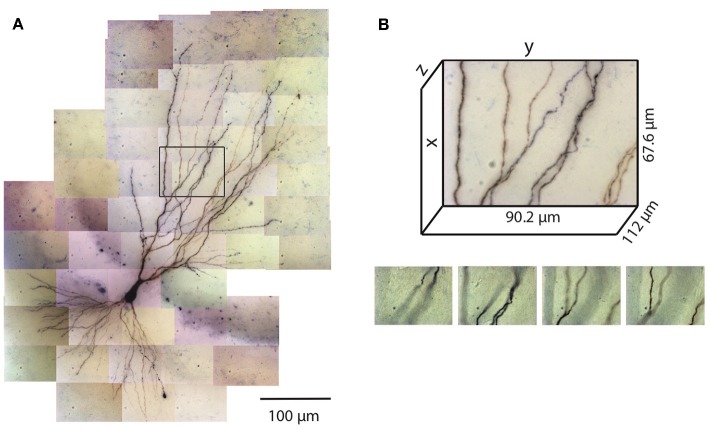
Tiles of tiff stacks covering the entire neuron. **(A)** A mouse CA3 neuron imaged at 100× (biocytin fill, dark reaction product, bright-field microscopy, NA 1.4). There are 51 tiles covering the entire neuron. 2D projection is shown. **(B)** Dimensions of one tile. Each tiff stack consists of 224 planes of images. The distance between successive image planes is 0.5 μm. Four planes at different depths in the tiff stack indicated by the black rectangle in **(A)** are shown below.

ShuTu uses tiles of tiff stacks covering the entire neuron (Figure 1). Nearby tiles should overlap by ~20%, in order to facilitate accurate stitching of tiles into a single image. We imaged hippocampal neurons using a Zeiss AxioImager microscope with AxioCam and ZEN blue software. Once the boundary in the field of view (*xy*) and the range of the depths (*z*) that contain the neuron were set, the images at each tile position and depth were acquired automatically, and the positions of the these images were stored in an xml file (image metadata). Other microscope/software combinations can be used, as long as tiff stacks and their relative positions are provided to ShuTu (see Materials and Methods for details). It is also possible to use ShuTu to reconstruct neurons imaged using two-photon, confocal, or wide-field fluorescence microscopy (see Discussion). However, we focus our description of the software mainly to neurons stained using biocytin and a dark reaction product.

The number of images required to capture the full three-dimensional (3D) morphology of a neuron depends on its size and the magnification of the microscope objective. The CA3 pyramidal neuron reconstructed here was relatively large and we imaged it using 100× objective (NA 1.4) (Figure 1). Therefore a total of 51 tiles were required, with 224 images per tile (0.5 μm increments through the depth of the slice), thus yielding a total of 11,424 images. This neuron is contained in a volume of ~400 × 600 × 100 μm^3^ and has a total dendritic length of ~8,800 μm. The full imaging process for the CA3 pyramidal neuron took ~2 h on the Zeiss microscope system we used. Faster imaging times (and fewer tiles) can be accomplished using lower magnification objectives, but in our experience 100× provides more accurate estimates of diameters for small-caliber dendrites. During imaging, care was taken to ensure that the microscope settings were optimized to obtain images of all dendrites, including those with the smallest diameter. This resulted in significant background noise, which was removed automatically in a final step of the reconstruction process (see below). We made no attempt to image or reconstruct axons, as these were of finer caliber than dendrites and for many neurons they were difficult to discern beyond a short distance from their origin near the soma.

### Image Processing

Because the ZEN blue microscope software provides individual image files in each tile, ShuTu first converts the image files into tiff stacks using the image metadata file (xml) and parsing the file names for depth information. Each tile was imaged successively through the depth of the slice, so no alignment of the images is required to form a stack. As each stack consists of 224 images, about 5 min of CPU time was required for each stack (see Materials and Methods for the system used). The CA3 pyramidal neuron reconstructed here consists of 51 tiles, and creating the stacks required a total of just over 4 h. With multiple CPU cores and sufficient memory, ShuTu can automatically distribute the task across multiple cores in parallel, resulting in approximately linear reduction in the real time required to construct the stacks.

After the tiff stacks are created, the tiles need to be stitched to find precise relative positions between the tiles. ShuTu also accomplishes this task in a parallel manner, requiring a similar amount of computational time as construction of the stacks. These two image processing steps are performed in series, but they can be executed sequentially without user intervention. In the case of our example CA3 pyramidal neuron, both of these steps were performed in just a few hours by using multiple CPU cores.

### Automated Reconstruction

**Figure 2:**
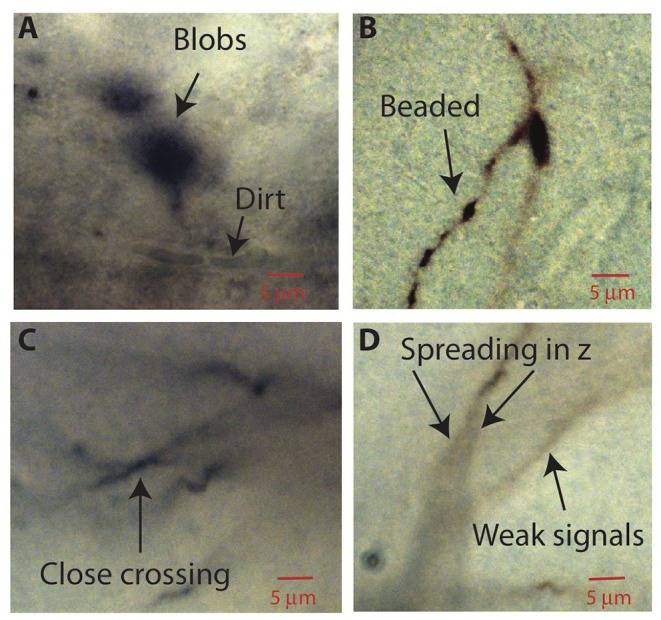
Aspects of bright-field images of biocytin-filled neurons that make automatic reconstruction challenging. Parts of images in single planes of the tiff stacks are shown. **(A)** Biocytin spills can create spurious signals. Dirt or dust can also add noise. **(B)** Thin branches can be broken into “beads.” **(C)** Close crossing between adjacent neuron branches. **(D)** A branch can cast bifurcating shadows in *z* with darkness level comparable to weak signals from nearby faint branches.

After image processing, ShuTu produces a draft reconstruction of the neuron using an automatic reconstruction algorithm (Materials and Methods). We devised the algorithm to specifically deal with several challenges posed by the bright-field images of biocytin-filled neurons (Figure 2). One is background noise (Figure 2A). While patching a neuron, biocytin can spill out and create blobs in the image stacks. Dirt or dust can be picked up, resulting in structures that share some features of neurites, especially as color information is not used. Second, during the process of fixing the tissue, thin dendrites can become beaded, with very faint signals between the beads (Figure 2B). Third, close crossings of adjacent branches require special attention to resolve (Figure 2C). Fourth, shadows of out-of-focus branches can be as strong as signals from thin dendrites in focus (Figure 2D), making it hard to trace some dendrites without being fooled by the shadows. These challenges make it difficult to create a perfect reconstruction from automated algorithms. Our algorithm was designed to address many of these issues, but some manual correction is nevertheless required. In the following, we outline the steps involved in the algorithm, using the tile shown in Figure 1B as an example. Technical details of the algorithm are presented in Appendix 2, which should be useful for adjusting the parameters for specific situations encountered by users.

#### Conversion to Gray Scale and 2D Projection

**Figure 3:**
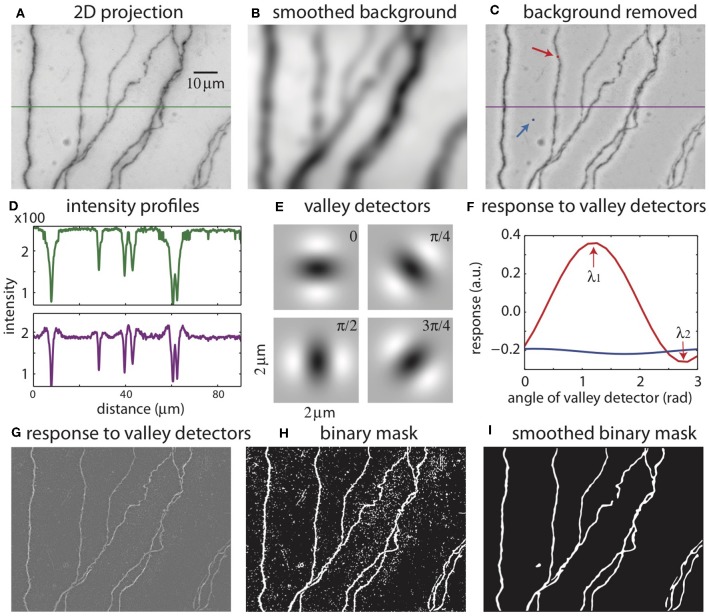
The process of creating a binary mask from 2D projection. **(A)** 2D projection from the image stack. The intensity profile across the green line is shown in **(D)**. **(B)** Smoothed background obtained from Gaussian smoothing of the 2D projection. **(C)** 2D projection after removing the smoothed background. The intensity across the purple line is shown in **(D)**. The red and blue arrows indicate the points to be tested with valley detectors in **(F)**. **(D)** Intensity across the midline in the original 2D projection [green line in **(A)**] and after removal of the background [purple line in **(C)**]. **(E)** Images of valley detectors at four orientations. **(F)** Responses to valley detectors at varying orientations for two points shown in **(C)** (red point, red line; blue point, blue line). λ_1_ and λ_2_ are the maximum and minimum responses, respectively. **(G)** The maximum responses to the valley detectors (λ_1_) for all pixels. **(H)** The binary mask obtained from thresholding λ_1_. **(I)** The smoothed binary mask.

The color images are converted into grayscale images, and the pixel intensities are scaled so that the maximum is 1. A minimum intensity projection of the tiff stack is then created, which has the same dimension as a single 2D plane in the stack. The intensity at each pixel is chosen to be that of the darkest pixel among all pixels in the stack having the same *xy* position. This minimum intensity projection reveals all neurites in the tiff stack (Figure 3A), along with noise from the sources mentioned above. To remove smooth variations due to uneven lighting, the 2D projection is blurred by Gaussian smoothing (Figure 3B) and subtracted from the original 2D projection (Figure 3C). Additionally, this process makes faint branches nearly as visible as well-stained ones (Figure 3D); the inverse peaks corresponding to the branches in the intensity profile have more even heights after the background removal (purple curve) than before (green curve).

#### Binary Mask

The 2D projection is used to create a mask, which is a binary image with the white pixels indicating the neurites and dark pixels the background (Figures 3E–I). An accurate mask is crucial for our reconstruction algorithm. Considering the intensity as heights, the neurites in the original 2D projection can be viewed as valleys of dark pixels. To create the mask, we evaluate the possibility that each pixel in the 2D projection belongs to a valley. This is accomplished by comparing the local patch of image centered at the pixel with valley detectors of varying orientations (Figure 3E) (Frangi et al., 1998). A valley detector is a 2D image consisting of an oriented dark band flanked by two bright bands. The response of the detector is the sum of the products of the corresponding pixels in the detector and the local patch (Figure 3F). The response has a maximum (λ_1_) at one orientation, and a minimum (λ_2_) at the orthogonal orientation (Figure 3F). If the local patch is nearly uniform in intensity, the response is close to zero at all orientations, and λ_1_ is small (Figure 3F, blue curve, which describes the responses at the blue pixel in Figure 3C). In contrast, if the local patch contains a valley, the maximum response (λ_1_) is large and the minimum response (λ_2_) is small (Figure 3F, red curve, at the red pixel in Figure 3C). If the patch contains a crater corresponding to a blob, λ_1_ can be large, but so can λ_2_, because there is no privileged orientation. These features are used to select pixels in valleys but not in blobs or in the background by thresholding λ_1_ while also factoring in the difference between λ_1_ and λ_2_, creating the binary mask (Figure 3H). The mask is further smoothed to eliminate noisy speckles and rough edges in the boundaries, creating the smoothed mask (Figure 3I).

#### SWC Points

We use the “SWC” format for representing the neuron morphology (Cannon et al., 1998). It consists of a list of SWC points. An SWC point is defined by six values: ID, branch type, *x*-position, *y*-position, *z*-position, radius, and the ID of the parent SWC point (-1 if there no parent). ID is an integer unique to the point. The value for branch type can be 1 for soma; 2 for axon; 3 for basal dendrite; and 4 for apical dendrite. *x, y, z* specify the position of the point, which should be on the center line of the branch; and radius specifies the radius of the branch at that position. The parent ID gives the connections between the SWC points. An SWC point and its parent specifies a cylindrical trapezoid volume that represents the small segment of the branch between the positions of the two points.

**Figure 4:**
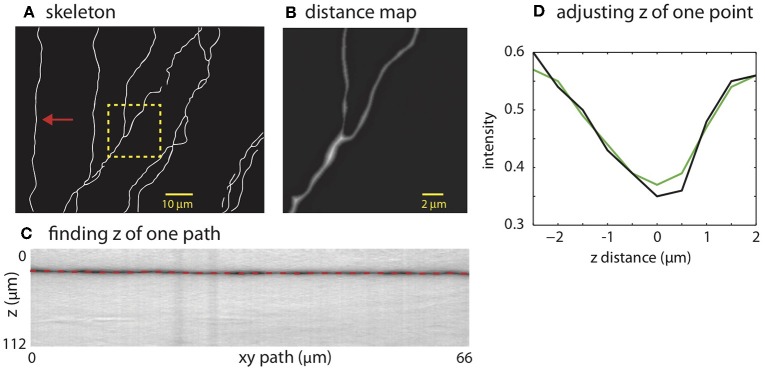
Creating SWC structure from the mask. **(A)** The skeleton obtained by thinning the mask. **(B)** Distance map computed from the mask. The square region highlighted in **(A)** is shown. The brightness of each pixel is proportional to the distance to the nearest boundary in the mask. **(C)** Finding the depth of the path. The image is constructed by cutting through the stack in *z*-dimension following the *xy* path indicated by the arrow in **(A)**. The dark band is the neurite along the path. The dotted line is the depth (*z*) computed using the left-right shortest path algorithm. **(D)** The depth of a candidate SWC point is further adjusted using the intensity profile in *z* at the *xy* position of the candidate point (black line). The point of minimum intensity in the smoothed profile (green line) is set as the depth of the candidate point. The *z* distance in the graph is relative to the *z* position of the end point.

The mask is used to place SWC points along the neurites. The SWC points are placed along the centerlines of the binary mask (Figure 4A). The radii of the SWC points are computed as the shortest distance from the positions of the SWC points to the nearest boundaries of the binary mask (Figure 4B). To determine the depths of the SWC points in the original tiff stack, we dissect the centerlines into segments between end points and/or crossing points. These segments are called “xy-paths” (e.g., Figure 4A, red arrow). Cutting through the tiff stack while following an xy-path, we create a “z-image” for that segment (Figure 4C). This z-image contains all pixels in the tiff stack whose *xy* positions lie in the xy-path. The branch whose 2D projection falls on the xy-path manifests as a dark valley in the z-image spanning from the left edge to the right edge (Figure 4C). ShuTu finds the line through the dark valley (red dotted line in Figure 4C), from which the depths of the neurites (and the SWC points) are determined. The distance between successive SWC points is set to roughly the sum of their radii. The distance is made shorter when the radii changes rapidly along the centerlines to reflect large changes in short distances in the dendritic morphology.

Invalid SWC points are automatically removed (see below regarding validity of SWC points), and the *z* of a valid SWC point is further adjusted to the nearby depth of minimum of intensity in *z*-dimension (Figure 4D). Adjacent SWC points along one xy-path are connected. If the removal creates a large distance between two consecutive SWC points, they are not connected. Biologically, sharp turns in neurites are rare. Therefore, to safeguard against possible errors, we do not connect SWC points if doing so creates sharp angles in consecutive lines of connections. To avoid connecting branches far away in depth, SWC points are not connected if the difference in *z* is too large. These decisions depend on parameters set by the user (Appendix 2).

#### Validity of SWC Points

**Figure 5:**
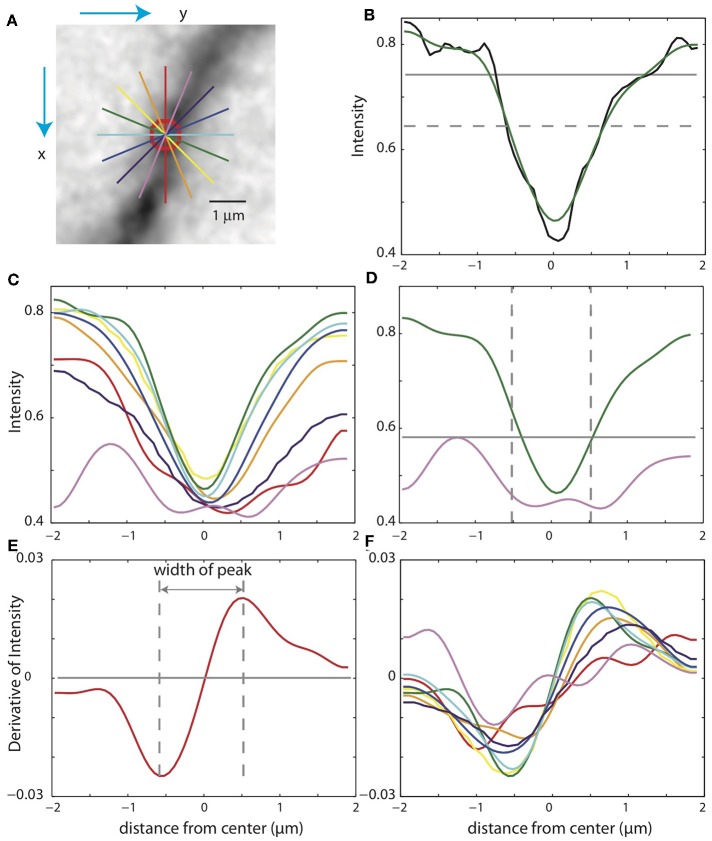
Checking the validity of an SWC point. **(A)** A patch of image around an SWC point to be examined. The image is taken from the *z*-plane of the SWC point. Profiles of the intensities along eight directions are taken (straight lines; colors indicate angles). The green line is the profile chosen to adjust the SWC point. The red circle indicates the radius of the SWC point. **(B)** The profile (black, raw; green, smoothed) along the green line in **(A)**. The dotted gray line is the baseline, and the solid gray line is the threshold. An inverse peak is judged valid if the flanks of the smoothed profile go above the baseline, and the minimum value goes below the threshold. **(C)** Smoothed profiles for all eight directions. **(D)** The chosen profile (green) and the profile at the orthogonal direction (violet). The vertical lines are at the half radius points. Note that the center of the profiles are slightly shifted compared to those in **(C)**. For the SWC to be valid, the minimum intensity of the profile at the orthogonal direction must be below the threshold (gray line) within the vertical lines. **(E)** Smoothed derivative of the smoothed profile in **(B)**. The vertical lines indicate the local maxima of the derivatives. The distance between the vertical lines is the width of the peak. **(F)** Smoothed derivatives of the profiles for all eight directions. The profile with the minimum width is chosen.

In some cases, the xy-paths from the centerlines of the binary mask are incorrect. For example, nearby branches can be merged in the mask. Checking the validity of the SWC points is thus crucial for eliminating mistakes. To do so, we take a square patch of the image centered at an SWC point in the plane of the point's depth. The size of the patch is set to four times the radius of the SWC point or 4 μm, whichever is greater. To reduce the possibility that a tilt in the intensity across the patch might interfere with the check, we subtract a linear fit to the intensity and scale the result to the original intensity range. We then create intensity profiles in eight directions centered at the SWC point (Figure 5A). For each profile, we look for a significant inverse peak after smoothing the profile (Figures 5B,C). The significance is checked against the baseline and fluctuations in the intensity. The baseline is set to the top 20% intensity value in the patch, and a parameter σ = 0.03 is used to characterize the fluctuations. A threshold, set to the half point between the maximum and minimum of the smoothed profile (Figure 5B, dotted gray line), is used to judge whether the smoothed profile has two flanks. Another threshold, set to the baseline minus 2σ, is used to judge whether the inverse peak is deep enough (Figure 5B, gray line). If both criteria are met, the profile is judged to have a significant inverse peak. The width of the inverse peak is the distance between the steepest descending point and the steepest ascending point of the peak, identified by the derivatives of the smooth profile (Figures 5E,F).

If none of the profiles have a significant inverse peak, the SWC point is invalid. Otherwise, we chose the profile with the minimum width among the valid ones. In some cases, an SWC point can be at the edge of thick dendrite or soma (see below). To eliminate them, we check wether the intensity within the half radius of the SWC point is low enough (Figure 5D). Specifically, we check that the intensity values of the smoothed profile (Figure 5D, violet curve), orthogonal to the chosen profile (Figure 5D, green curve) within the half radius (Figure 5D, dotted vertical lines), is smaller than a threshold. This threshold is set to the maximum of the chosen profile within the range plus σ. If not dark enough, the SWC point is invalid.

If the SWC point passes the validity test, we set its radius to the half width of the inverse peak in the chosen profile. Its *xy* position is adjusted to that of the inverse peak, and *z* position is adjusted to the depth of the nearby intensity minimum in *z* (Figure 4D). To ensure that this adjusting process converges, we adjust each SWC point three times iteratively. If the final *xy* position shifts from the original position more than twice of the original radius, we mark the SWC point invalid since it is most likely created erroneously. Finally, if the final radius of the SWC is smaller than 0.2 μm or larger than 10 μm, the SWC is most likely due to noise and is marked invalid.

#### Mark Pixels Occupied

**Figure 6:**
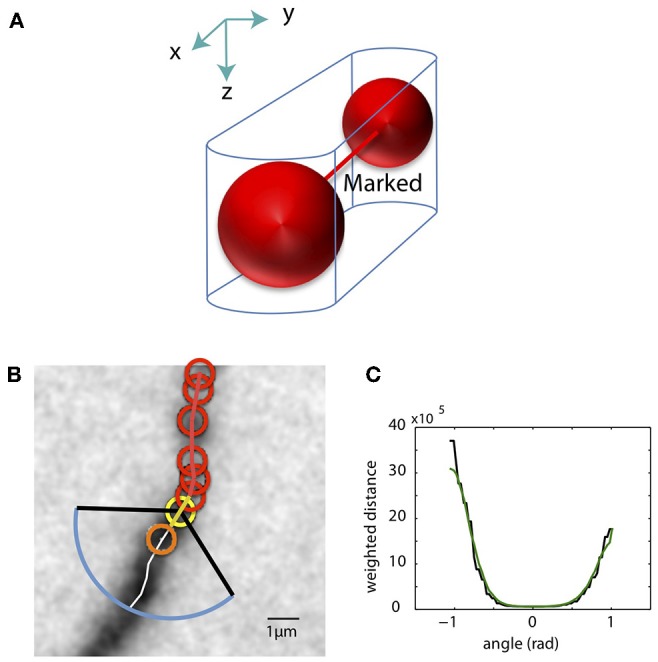
Mark pixels as occupied and extending SWC structure in 3D. **(A)** Mark pixels in tiff stack as occupied. The pixels around two connected SWC points (red spheres), formed by two half cylinders and a trapezoidal prism, are marked as occupied. **(B)** The candidate point for extension is searched in the plane of an end SWC point (yellow circle). Red circles are the SWC points that are connected to the end point. The search is done along a shortest distance path running through the neurite (white line). This path is determined by building the intensity-weighted shortest distance profile along an arc (blue line) enclosed by two black lines. The valid SWC point closest to the end point is selected as the new SWC point (orange circle). **(C)** The profile of the shortest distances along the arc [blue line in **(B)**]. The green line is the smoothed version. The angle is measured relative to the line connecting the end point to its connected SWC point [yellow line in **(B)**]. The minimum position in the smoothed profile is selected as the starting point of the shortest path shown in **(B)**.

As the SWC points are created, we mark pixels in the tiff stack in the vicinity of the SWC points as occupied. The pixels around two connected SWC points, formed by two half cylinders and a trapezoidal prism, are marked as occupied (Figure 6A). Before creating a new SWC point, we check whether its center point is marked as occupied; if so, no SWC point is created. This avoids creating redundant SWC points for the same piece of dendritic branch.

#### Thick Dendrites and Soma

The widths of dendrites can vary several-fold from the thin terminal dendrites to the thick apical dendrite near the soma. The thick dendrites and the soma can be missing from the binary mask, which is created with the valley detector tuned for detecting thin dendrites. Therefore, only edges of the thick dendrite and soma are captured in the mask, leading to invalid SWC points that are eliminated. To solve this issue, we separately detect the presence of thick dendrites and soma. The thick dendrites and soma are typically well-stained and show up as the darkest locations in the 2D projection. We use this fact to decide whether there are thick dendrites and soma that are not covered by existing SWC points. If the lowest intensities in the pixels covered by the existing SWC points are brighter than the lowest intensities in the 2D projection, we decide that the binary mask missed the soma or thick dendrite. We create a binary mask on a 2D projection, excluding pixels around the existing SWC points, by thresholding the pixel intensities of the 2D projection. New SWC points are added based on this new mask.

#### Extending SWC Points in 3D

The SWC structure created with 2D projections can contain errors. Typically the binary masks can be incomplete or incorrect in some parts due to weak signals, occlusions produced by branch crossing, or mergers of closely parallel branches. This leads to gaps in the SWC structure representing continuous dendritic branches. To bridge these gaps, we extend the SWC points in 3D (the tiff stack) from the end points in the SWC structure.

To minimize interference from noise, we first delete isolated SWC points that are not connected to any other SWC points. We then mark pixels near the existing SWC points as occupied (Figure 6B, red circles) to ensure that the extension does not create duplicate SWC points.

From an end SWC point (Figure 6B, yellow circle), we search for the next candidate SWC point. We draw an arc of radius 3 μm or twice the radius of the end point, whichever is greater, in the plane of the end point (Figure 6B, blue arc). The arc spans from −π/3 to π/3 (Figure 6B, black lines) relative to the line from the end point to its previously connected SWC point (Figure 6B, yellow line). The shortest intensity-weighted distances from the points on the arc to the end point are computed (Figure 6C, black line). With the smoothed profile of the distances, the point with the minimum distance is selected (Figure 6C, green line), and the shortest distance path from this point to the end point is found, which should follow along the neurite (Figure 6B, white line). The depth of the neurite is found using the xy-path technique (Figure 4C) along the shortest distance path.

The candidate SWC point for extension (Figure 6B, orange circle) is placed on the shortest distance path, starting from the end point marching toward the arc. We test the validity of the candidate SWC point, during which the *xy* position and radius are adjusted. To cover weak branches, the test is made less stringent by accepting shallower inverse peaks in the intensity profiles used for the test (Figures 5B,C). If accepted, the extension process continues from the new SWC point as the end point. If the candidate point is marked occupied, the extension stops, and the possibility of connecting the end point to the existing SWC points that marked the occupation are evaluated (see below). The extension stops if the test fails for all points along the shortest distance path.

#### Connecting Broken Segments

After extending SWC points in 3D, a continuous branch can still be represented with broken segments of SWC points, especially if the underlying signal is broken or there are closely crossing branches (e.g., Figures 2B,C). We connect these segments with heuristic rules based on the distances between the end points, in order to recover the branch continuity (Appendix 2). After connecting the end points, the SWC structure for the tiff stack is complete.

**Figure 7:**
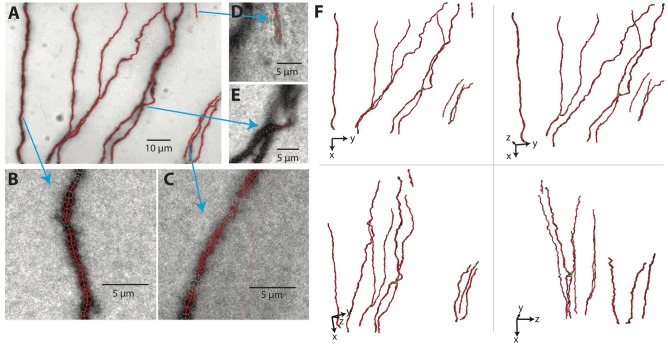
The SWC structure overlaid on the image. **(A)** The SWC structure projected on to the image of 2D projection. Red circles are SWC points. Connections between them are indicated with red lines. **(B–E)** SWC structure overlaid at the specific planes in the tiff stack, zoomed in to show more details. Arrows indicate the corresponding regions in the 2D projection. **(F)** The SWC structure viewed from four different angles to reveal the 3D structure. The viewing angles are indicated with the directions of *xyz* coordinates.

The results for our example tiff stack are shown in Figures 7A–E, in which the SWC points are overlaid with the underlying image, and in Figure 7F, in which the SWC structure is shown from four different view angles in 3D to reveal more details. For this particular tiff stack, the automated reconstruction is mostly accurate, except that an elongated piece of dirt is mistaken as a neurite, and a close crossing of two branches is incorrectly connected. These errors need to be corrected manually (see below).

#### Subdivision in *z*

The 2D projection can be complicated when there are many branches in one tiff stack, which often leads to missed branches due to occlusions. One way of mitigating this problem is to divide the tiff stack in *z* into several slabs with equal heights in *z*. SWC points are created separately for each slab as described above, and then combined for the entire stack. Extension from the end points is done with the entire stack. When branches extend across the boundaries between subdivisions of tiff stacks, they are automatically connected by extension from the end points, as described above.

ShuTu allows the user to decide how many subdivisions (slabs) are necessary based on the complexity of the morphology and the thickness of the tiff stacks. The user should keep in mind that a large number of subdivision slows down the automated tracing. In our example neuron, we divided all tiff stacks into eight slabs.

#### Combining SWCs

**Figure 8:**
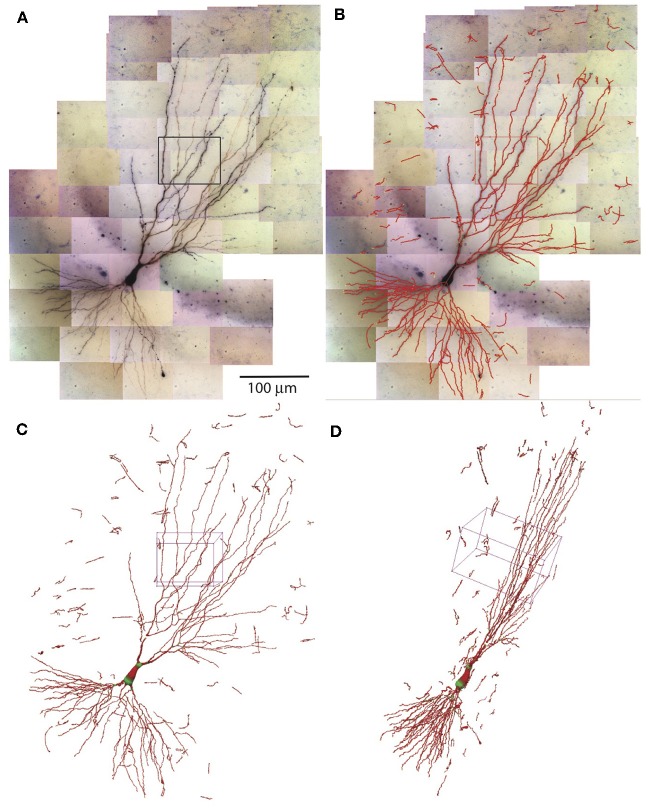
Combining SWCs from the stacks for the entire neuron. **(A)** 2D projection of the entire neuron. Individual tiff stacks are stitched together to obtain their relative coordinates. The stack in Figure 7 is highlighted with a black rectangle. **(B)** The SWC of the entire neuron is obtained by combining the SWCs of individual tiff stacks. The SWC points are overlaid onto the 2D projection. **(C)** 3D view of the SWC structure. The 3D box corresponds to the highlighted stack in **(A)**. **(D)** The 3D view from a different angle.

The SWCs determined in individual tiles of tiff stacks are combined to form the SWC of the entire neuron. The positions of SWCs are shifted based on the relative coordinates obtained in the stitching process. The SWC points of individual stacks are read in sequentially. To avoid duplicated SWC points in the overlapping regions of adjacent stacks, pixels near the SWC points that are already read in are marked occupied. If the position of SWC points are at the marked pixels, they are deleted. After reading in the SWC points of all stacks, we extend the end points and connect them if they are nearby. Isolated short branches (<20 μm) and small protrusions (<5 SWC points) from main branches are deleted to reduce noise in the SWC structure. The resulting SWC structure for the example neuron is shown in Figures 8A–D.

The entire process of automated reconstruction of the example neuron took about 4 h on our basic desktop system using three CPU cores (see Materials and Methods for the system specs). With more powerful computers, the time can be further reduced approximately linearly with the number of CPU cores used.

### Manual Editing and Error Correction

The SWC structure created by the automatic algorithm requires editing, such as removing noise, tracing thin or faint dendrites, connecting ends, and correcting mistakes in the radii and positions of the SWC points and in the connections between them. We have designed ShuTu to make these operations easy for the user. In this section we highlight a number of editing techniques.

#### Inspecting the Reconstruction

**Figure 9:**
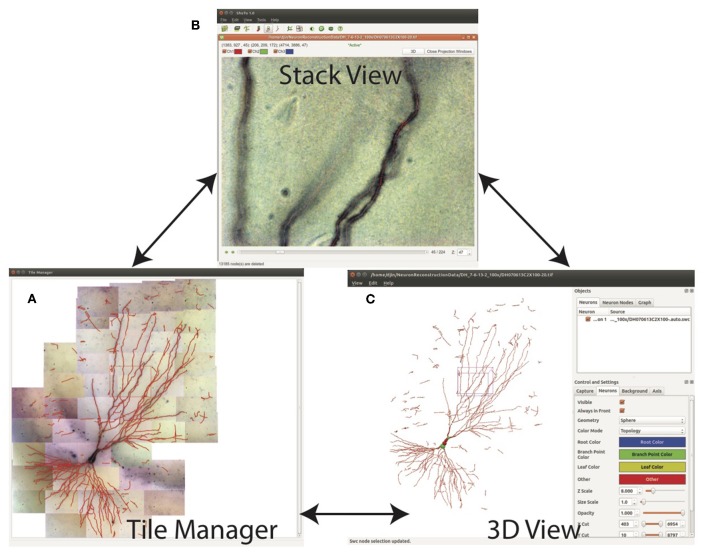
Three views for examining the SWC structure. **(A)** Tile manager. 2D projection of the stitched stacks is superimposed with the 2D projection of the SWC structure. **(B)** Stack view. One stack is loaded, with the SWC points in the stack overlaid onto the image. The 2D projection view of the stack can be created within this window. **(C)** SWC view. The 3D structure can be viewed from different angles and edited.

The SWC structure can be examined in three modes: Tile Manager, Stack View, and 3D View (Figure 9). In Tile Manager, the SWC structure is overlaid with 2D projection of the entire neuron (Figure 9A). In this view, it is easy to identify missing, discontinuous, or incorrectly connected branches.

Double clicking on one tile in Tile Manager loads the tiff stack into Stack View (Figure 9B), in which the SWC structure is overlaid with the image. The radii, depths, and connectivity of the SWC points can be examined in detail by scrolling up and down through the *z* dimension of the tiff stack.

**Figure 10:**
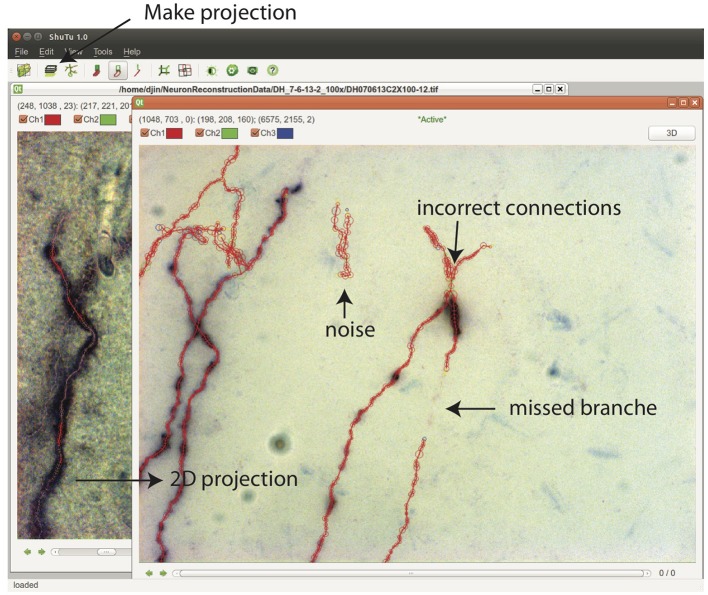
In the Stack View, clicking on the Make Projection button creates the 2D projection of the tiff stack and the SWC structure. It is easy to spot mistakes in this view. SWC points can be removed and their properties changed. The connections between SWC points can be modified. Selecting one SWC point and pressing z locates the points in the Stack View for further examination and modification.

From Stack View, a 2D projection can be created by clicking on the Make Projection button (Figure 10). There is an option to subdivide the stack into multiple slabs in *z*, in which case separate 2D projections are created. Subdivision is useful when the branching patterns are complicated. Mistakes in the reconstruction can be easily spotted in Projection View, including missed branches, broken points, incorrect connections, and inclusion of noise (Figure 10). Incorrect positions and diameters for the SWC points are easy to identify as well.

In 3D View, the SWC structure can be rotated and shifted in order to reveal incorrect connections, especially large jumps in *z*, which can be obscure in other views.

Editing can be done in Stack View, Projection View, and 3D View. In all cases, after any editing, the SWC structure is updated in all views. A selected point can be deleted or moved and its radius can be modified. A selected point in Projection View or 3D View can also be located in Stack View for further examination and modification using the tiff stack.

#### Adding SWC Points

**Figure 11:**
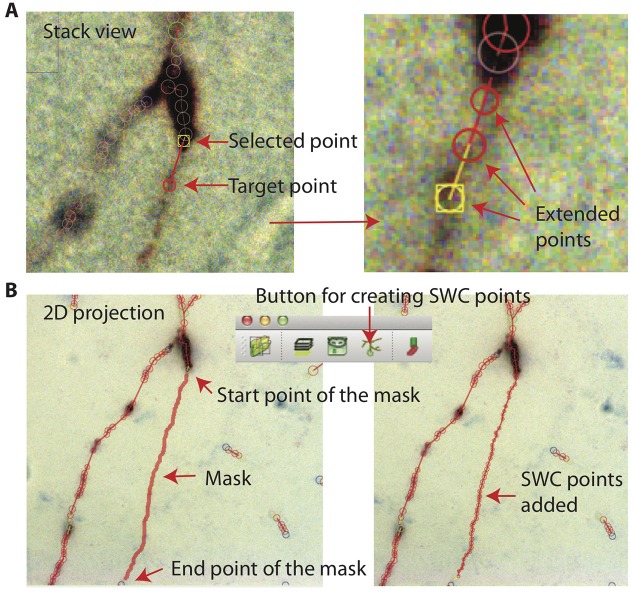
Creating SWC points. **(A)** In Stack View, an SWC point is selected. Find the target point by finding the focus plane of the branch. Clicking on the target point creates SWC points connecting the target point to the selected point along the branch. **(B)** In Projection View, pressing r starts mask creation. Click on the starting point and Shift-click on the end point along a branch creates a mask. Clicking on the Mask → SWC button creates SWC points along the mask.

In Stack View, SWC points can be added in three ways. The first method is smart extension. The user selects an SWC point on a branch that needs extension, finds a target point on the branch and locates the focus plane in *z*, and then clicks on the target. SWC points will be added along the branch from the selected SWC point to the target point (Figure 11A). The path is computed with the shortest distance algorithm, and the radii and positions of the SWC points are automatically calculated using the automated algorithm described above. The second method is manual extension. It is the same as the smart extension, except that the only point added is at the target point and its radius needs to be adjusted manually. The third method is mask-to-SWC (Figure 11B). In Projection View, a mask along a branch is drawn by selecting the start and end points. The path is automatically computed with the shortest-distance algorithm. The mask can also be drawn manually. After the mask is completed, it is converted to SWC points along the branch. The positions and radii of the SWC points are computed automatically.

These three ways of adding SWC points are complimentary. When the branch to be reconstructed is long, the mask-to-SWC method is efficient. However, it requires that the underlying signal is strong enough, otherwise the computation of the path and the depths can be inaccurate. When the branch to be covered is short, the smart extension method is efficient, although it also requires a relatively strong signal. Manual extension always works.

ShuTu users can reconstruct the entire neuron with one of these three methods. The extension methods can be used after creating a single seed SWC point. However, the process is tedious because the focus plane must be located in every click. The mask-to-SWC method traces branches in 2D projections, and is therefore more efficient.

#### Modifying Connections

The end points in the SWC structure are highlighted with blue or yellow colors. In some cases, it is necessary to connect nearby points that have been incorrectly identified as end points. This can be done by selecting two end points and connecting them. If the distance between the two points are more than the sum of their radii, SWC points can also be added automatically while bridging the gap. A selected end point can also be automatically connected to its nearest neighbor.

Making connections between two points are denied if they are already connected. If this happens to two nearby points that the user would like to connect, the user should look for a “loop” in the SWC structure connecting these two points due to incorrect connections made elsewhere. Selecting the two points highlights the loop. After breaking the incorrect connections, the two points can be connected.

Incorrect connections can be broken after selecting two connected SWC points. The branching points are highlighted with green; these points need to be examined carefully for incorrect connections, especially when branches cross.

**Figure 12:**
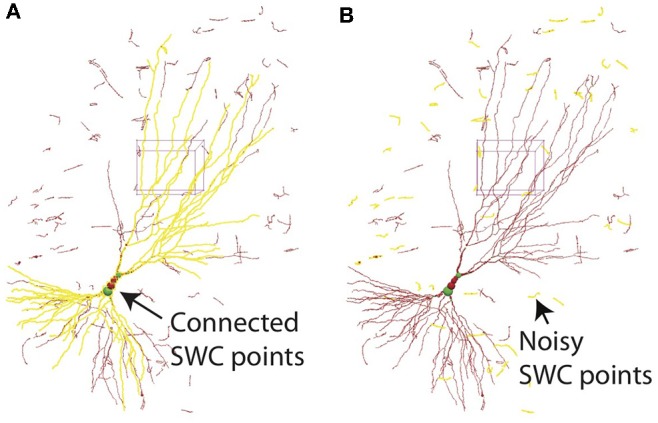
**(A)** In 3D View, selecting one SWC point and pressing h-5 selects all SWC points connected to the selected. This operation is useful for detecting broken connections. **(B)** Pressing h-7 selects all branches with total length smaller than a chosen threshold. This operation is useful for deleting noise.

All SWC points connected to a selected point can be highlighted (Figure 12A). This is useful for finding broken connections in the SWC structure. Structures with short total length can also be selected (Figure 12B), and can be deleted with a single command. This is useful to reduce noise in the automated reconstruction. At the end of the reconstruction, all SWC points that belong to the neuron should be connected. At this point, all remaining points (presumably noise) can be deleted by selecting all connected points in the neuron and deleting the unselected points with a single command.

### Reconstruction Efficiency

**Figure 13:**
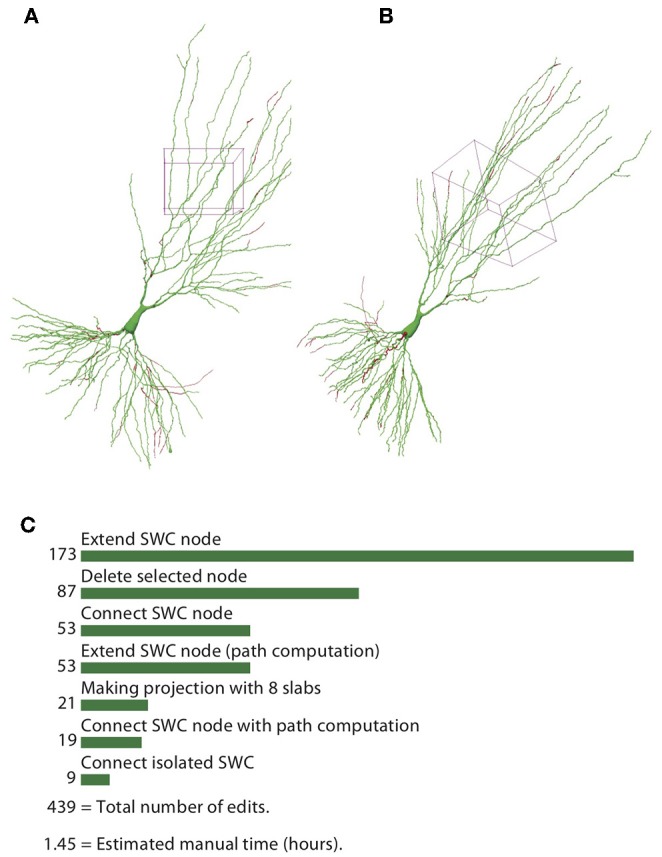
Reconstructed neuron after editing. **(A,B)** Two different views of the reconstructed neurons. The SWC points from the automatic reconstruction are in green, and those added in the editing process are in red. **(C)** Top operations done in the editing process and the total number of edits.

To quantify the efficiency of reconstructing neurons through the automatic algorithm and manual editing, we counted the number of editing operations (NEO) required for achieving the final reconstructions, starting from the one generated by the automatic algorithm. The results for the example neuron are shown in Figure 13. The SWC points that are added in the editing phase are shown in red, and those from the automatic reconstruction are shown in green (Figures 13A,B). The added SWC points are about 5% of the total SWC points in the structure. The NEO is 439. Among the editing operations, extensions are dominant. Correcting connection mistakes are sizable as well. The manual time spent in repairing the automatic reconstruction was around 1.5 h.

**Figure 14:**
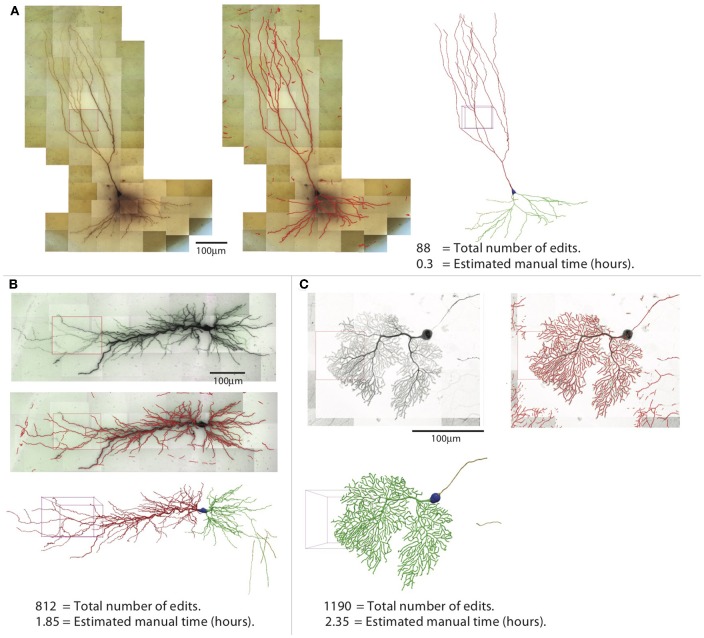
Three examples of reconstructions. Shown for each neuron are the 2D projection of the images, automated reconstruction on top of the 2D projection, and the final reconstruction (blue, soma; red, apical dendrite; green, basal dendrite; gold, axon). **(A)** A pyramidal neuron in the mouse CA3 region imaged at 100× (biocytin). **(B)** A pyramidal neuron in the rat CA1 region imaged at 63× (biocytin). **(C)** A mouse Purkinje cell imaged at 63× with confocal microscope (fluorescence).

The efficiency of reconstruction depends on the image quality and the complexity of the neuron morphology. For neurons with sparse processes, the automated reconstruction captures most of neuronal structure, and manual editing is not intensive. For the example shown in Figure 14A, which is another mouse CA3 pyramidal neuron, simpler than the one shown in previous figures, the NEO is 88, and the time spent in editing was ~20 min. In contrast, when the processes are dense, the automated reconstruction contains many omissions and mistakes, so manual editing takes more effort. An example is shown in Figure 14B, which is a rat CA1 pyramidal neuron; the NEO is 812, and the time spent in editing was ~1.9 h. Another example of a complex neuron is shown in Figure 14C, which is a mouse Purkinje cell labeled with fluorescent dye and imaged with Zeiss 880 confocal microscope at 63× magnification. The increased complexity decreases the quality of automated reconstruction; ~2.4 h of editing was required and the NOE is 1,190. This example also shows that our software is not restricted to tracing biocytin-labeled neurons.

### Comparison to Other Algorithms for Automatic Reconstruction

To assess the performance of our automated algorithm relative to other algorithms, we compared automatic tracing results of three approaches: our own, neuTube (Zhao et al., 2011; Feng et al., 2015), and Vaa3D (more specifically, Vaa3D-APP2) (Xiao and Peng, 2013). Among many available automatic reconstruction algorithms (Acciai et al., 2016), we selected neuTube and Vaa3D for comparison because they are widely known and adopted by the community.

We selected a tile covering part of the basal dendrites in the biocytin-filled neuron shown in Figure 1. Since neuTube and Vaa3D are designed for tracing dark-field (fluorescent) images, we converted the original bright-field image to a dark-field image. We tested several preprocessing methods for this purpose, including directly inverting the image intensities, local background subtraction provided by Vaa3D plugins (Peng et al., 2010), and adaptive image enhancement developed for neuron tracing (Zhou et al., 2015). We found that only the local background subtraction method, which computes the background of any given location by averaging its neighboring samples within a certain range, produced acceptable automatic reconstructions when the preprocessed image was supplied to neuTube or Vaa3D. In each program, we tuned adjustable parameters in the open-source code to obtain the best results.

**Figure 15:**
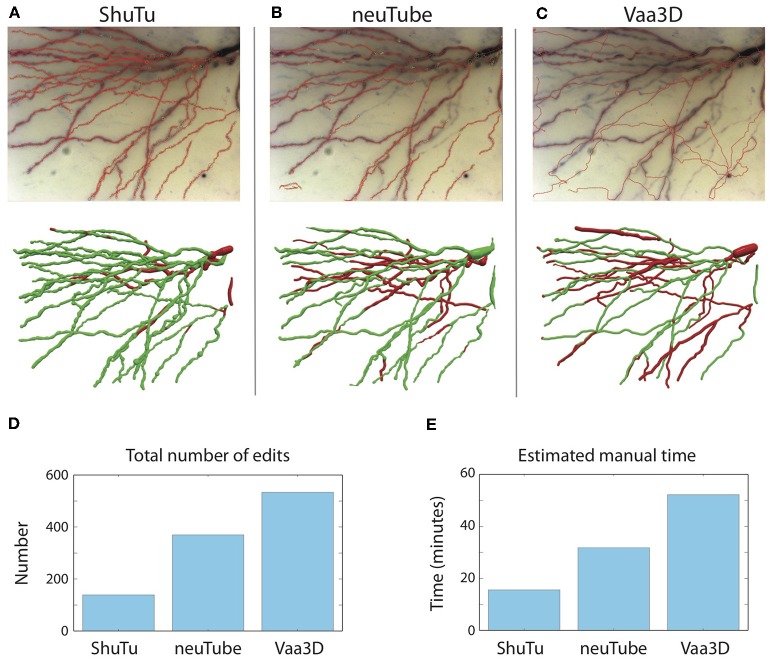
Comparisons of automated reconstructions from ShuTu, neuTube, and Vaa3D. **(A)** Automated reconstruction generated by ShuTu on one image tile (red circles) overlaid on the 2D minimum-intensity projection of the tile (top). The tile covers part of the basal dendrites of the neuron shown in Figure 1. The SWC structure after manual editing (below). Green indicates the SWC points automatically generated; red indicates those added during the editing. **(B)** Automated reconstruction generated by neuTube. **(C)** Automated reconstruction generated by Vaa3D. **(D)** Comparison of the NEO (number of editing operations) for the three methods. **(E)** Comparison of the estimated manual editing time for the three methods.

As shown in Figure 15, our automated algorithm produced a reconstruction that required much less manual editing and corrections than neuTube or Vaa3D. Our automated reconstruction covered more neurites (Figures 15A–C). After manual editing and corrections, the percentages of SWC points created by the automated reconstructions and retained in the final reconstructions were 95% of the total SWC points for our algorithm, 72% for neuTube, and 70% for Vaa3D. The NEO values were 139, 370, and 534, respectively (Figure 15D); and the estimated manual time spent on editing and corrections were 17, 32, and 52 min, respectively (Figure 15E). For neuTube, most manual time was spent on manually tracing missed faint branches. For Vaa3D, besides the missing branches, additional time was required to delete erroneous traces (see Figure 15C); furthermore, the estimates of the branch radii were mostly far off, and adjusting the radii of the SWC points also contributed to the manual time. Comparisons of the automated reconstructions to a fully manual reconstruction of the tile further supported that our algorithm captures the dendrites more accurately than neuTube or Vaa3D (Supplementary Material).

## Discussion

We have demonstrated how ShuTu can be used to reconstruct neuron morphology by converting microscope images to structures described by a collection of SWC points. Our goal is to provide a practical system that can be readily implemented and used in labs who need accurate dendritic reconstructions of neurons, often studied and stained following recordings with patch-clamp electrodes. As an open-source software package, ShuTu can be continuously improved by the community. We have also provided raw images of the example neurons (Jin, 2019), which should be useful for testing and improving the software.

Software for automated reconstruction is often evaluated by comparing automated reconstructions to manual reconstructions (Peng et al., 2015; Acciai et al., 2016). We have not done this systematically, because of our assertion that all automated reconstructions, including those produced by ShuTu, are error prone. Our approach was to develop software that used automated algorithms only as an initial step to assist manual reconstruction and minimize the amount of editing time required by the user. The objective, therefore, is the same as manual reconstructions; namely reconstructions that can be considered “gold standard” (Peng et al., 2015), but are achieved in far less time than fully manual approaches.

To achieve this objective, we complemented our automated reconstruction algorithms with editing functions in ShuTu's graphical user interface (GUI). These functions improve the efficiency of editing, and provide a tool for counting the number of editing operations (NEO). Our software should be judged on its ability to produce gold standard reconstructions while minimizing NEO. For example, our automatic reconstruction algorithm can be aggressive in finding neurites, including faint ones. This introduces noise into the automated reconstruction (and thus apparently “bad” initial reconstructions), but the noise can be easily edited out after genuine dendritic branches are annotated (Figure 12).

There are many parameters in our automated reconstruction algorithm. Users should experiment with these parameters, as the optimal settings may depend on properties of the images, which are likely to vary depending on staining and imaging procedures. Among the most important parameters are the distances between pixels and between the successive planes, which are determined by the image acquisition process. Also important is the number of subdivisions of one tiff stack. Since our algorithm relies on 2D projections, subdivision reduces overlap of neurites from different depths, thus improving reconstruction quality. Checking the validity of each SWC point is also critical, so the users should pay close attention to adjusting these parameters. Appendix 2 describes other important parameters and technical details of the automatic reconstruction algorithm.

There are a number of open-source software packages for reconstructing neurons, most notably Vaa3D (Peng et al., 2014) and neuTube (Feng et al., 2015). Vaa3D has extensive capabilities for processing images from various sources (Peng et al., 2014). In contrast, we focused on optimizing our software for the particular application of neurons stained with a dark reaction product following patch-clamp recording. Although ShuTu can work for neurons stained in other ways (see Figure 14C), we have no attempt to optimize it for use with multiple staining and imaging procedures. In addition, ShuTu was developed with a philosophy that perfect automatic reconstruction is difficult, if not impossible. Therefore, we emphasized the importance of manual annotation and error correction. In keeping with this philosophy, ShuTu includes a user-friendly GUI to facilitate these processes.

Another open-source software package for neuron reconstruction—neuTube—also has a strong 3D capability for manipulating SWC structure (Feng et al., 2015). As the GUI of ShuTu is based on the GUI of neuTube (T. Zhao is a contributor to both), many of the features of ShuTu are adaptations of neuTube. However, we should emphasize that the algorithm used for automated reconstruction in ShuTu is novel and unrelated to neuTube. Additionally, ShuTu includes several important extensions of the GUI. NeuTube was designed to deal with a single tiff stack. As such, it can only be used for reconstructing neurons fully contained in a single tiff stack. ShuTu is a complete solution that includes the capability to deal with multiple tiff stacks, including modules for processing and stitching the images. In the interactive mode, the neuron structure is represented in multiples ways that are all linked (Figure 9), thus improving the ease and accuracy of editing the SWC structure.

Our automatic reconstruction algorithm is specifically designed for meeting challenges of tracing from images obtained from biocytin-filled neurons. For this purpose, it outperforms neuTube and Vaa3D (Figure 15). There are some caveats, however, as neuTube and Vaa3D were developed to trace neurons with fluorescent images. Although we preprocessed images to optimize the application of Vaa3D and neuTube to biocytin-filled neurons, there could be some other preprocessing methods that lead to better results. Also, the performance of the algorithms may strongly depend on images and neuron types. For comparing automatic reconstruction algorithms of neurons, a better approach could be something like DIADEM (Brown et al., 2011), in which the creators themselves tune the parameters for a common dataset and a diverse group of users edit them and measure NEOs on a suitable GUI platform, such as ShuTu.

Commercial solutions for neuron reconstruction also exist (e.g., Neurolucida 360, MBF Bioscience; Imaris FilamentTracer, Bitplane). Detailed comparison of ShuTu to these other software packages is difficult, as it requires mastery of all of them to be fair. We encourage authors and users of other software packages to test ShuTu on their dataset or test their favorites on the images used in this work. We welcome feedback from the user community.

Staining neurons with biocytin is common in patch-clamp experiments. However, methods for reconstructing neurons based on biocytin are limited. When dealing with bright-field images like the biocytin data, a common strategy is to apply some preprocessing method first (Narayanaswamy et al., 2011; Türetken et al., 2011; Zhou et al., 2015), making the images friendly for automatic reconstruction. Preprocessing, however, is often computationally intensive and does not guarantee good performance. ShuTu is specifically tailored to deal with inherent problems with images from biocytin filled neurons and does not require preprocessing.

ShuTu is not restricted to biocytin-filled neurons. We have shown that it can also handle images from confocal and fluorescent microscopy, simply by inverting the images. However, we made no attempt to optimize ShuTu for this application. For now, we have chosen to leave this enhancement to future iterations (by us or others) and instead optimize ShuTu for one common method of staining and imaging patch-clamped neurons.

Improving image quality will inevitably improve the efficiency and accuracy of neuron reconstruction. Users should ensure that high quality images are obtained. Tissue fixation and clearing processes can also influence the accuracy of the reconstructed neurons by causing tissue shrinkage or influencing image quality. To be accurate, these factors need to be quantified for specific experimental conditions and the dimensions of the reconstructed neurons need to be adjusted to account for shrinkage and distortion.

Accurate estimates of dendritic diameter are important for computational modeling of neurons (Anwar et al., 2014; Psarrou et al., 2014). Our automated algorithm works quite well in determining the diameters, as shown in Figures 7B,C. Manual adjustments of the diameters are rarely necessary during the manual editing stage. However, systematic biases can exist, especially if spines are densely stained and blurred in the images, for example in the Purkinje neuron shown in Figure 14C. Users need to account for these biases before using the SWC structures for simulations.

ShuTu has some limitations. It is not designed to trace axons, which are often too faint following biocytin staining in slices, and therefore difficult to trace automatically (and in many cases even manually). ShuTu also provides no mechanism for marking spines. It is possible that editing operations currently requiring human judgements, such as when dendritic branches closely cross each other, could be automated in the future using machine learning approaches (Turaga et al., 2010).

In conclusion, we have shown that ShuTu provides a practical solution for efficient and accurate reconstruction of neuron morphology. The open-source nature of the software will allow the research community to improve the tool further, and increased efficiency in neuronal reconstruction should facilitate more studies incorporating quantitative metrics of dendritic morphology and computer simulations of dendritic function.

## Materials and Methods

### Whole-Cell Recording and Neuron Staining

Acute hippocampal slices were prepared from mice and rats (17–30 days old). After animals were deeply anesthetized with isoflurane, they were decapitated and the brain rapidly removed into chilled cutting solution consisting of (in mM) 215 sucrose, 2.5 KCl, 20 glucose, 26 NaHCO_3_, 1.6 NaH_2_PO_4_, 1 CaCl_2_, 4 MgCl_2_, and 4 MgSO_4_. Hippocampi were dissected out and cut into 400 μm thick transverse sections on a Leica VT-1200S vibrating microslicer (Leica, Ltd., Germany). The cutting solution was slowly exchanged with artificial cerebrospinal fluid (ACSF) containing (in mM) 124 NaCl, 2.5 KCl, 10 glucose, 26 NaHCO_3_, 1.0 NaH_2_PO_4_, 2.0 CaCl_2_, and 1.0 MgCl_2_. The slices were incubated at room temperature for at least 1 h before recording, and then were transferred as needed to a submersion-type recording chamber perfused with ACSF at 2 ml/min. For rat hippocampal slices, the cutting solution contained (in mM) 204.5 sucrose, 2.5 KCl, 1.25 NaH_2_PO_4_, 28 NaHCO_3_, 7 dextrose, 3 Na-pyruvate, 1 Na-ascorbate, 0.5 CaCl_2_, 7 MgCl_2_, and the ACSF contained (in mM) 125 NaCl, 2.5 KCl, 1.25 NaH_2_PO_4_, 25 NaHCO_3_, 25 dextrose, 3 Na-pyruvate, 1 Na-ascorbate, 1.3 CaCl_2_, 1 MgCl_2_. 350-μm-thick slices were sectioned at an oblique angle. After 30–60 min of recovery in ACSF at 35–37°C, the chamber was maintained at room temperature. Both cutting and ACSF solutions were saturated with 95% O_2_ and 5% CO_2_ (pH 7.4).

Whole-cell recordings were obtained by visualized patch techniques under IR-DIC optics. The recording pipette resistance ranged between 4 and 6 *MΩ*. Series resistance (6 - 15*MΩ*) and input resistance were monitored throughout each voltage-clamp or current-clamp recording. Recordings with >10% change in series resistance were excluded. For mice, the intracellular pipette solution consisted of (in mM) 135 K-gluconate, 5 KCl, 1 CaCl_2_, 0.1 EGTA-Na, 10 HEPES, 10 glucose, 5 MgATP, 0.4 Na_3_GTP, and 0.1% biocytin, pH 7.2, 280–290 mOsm; for rats, the solution consisted of (in mM) 130 K-gluconate, 10 KCl, 10 Na_2_-phosphocreatin, 10 HEPES, 4 Mg-ATP, 0.3 Na-GTP, 50 μM Alexa Fluor 594 (Invitrogen, Waltham, MA), and 0.2% biocytin, pH 7.2, 295 mOsm. Resting potential ranged from −69 to −58 mV. Maximal recording time after dissection was 6 hr. Recording temperature was set to 32.0 ± 0.1 C° for mouse slices and to 33–35°C for rat slices using a TC-344A single-channel temperature controller (Warner Instruments, Inc, Hamden, CT, USA). All experiments were executed with a Dagan BVC-700A amplifier, digitized (3–5 kHz) using an ITC-16 analog-to-digital converter (Instrutech) or BNC-2090 and BNC-2110 boards (National Instruments, Austin, TX), and analyzed using custom-made software for IgorPro (Wavemetrics Inc., Lake Oswego, OR, USA). All chemicals were purchased from Sigma-Aldrich (St. Louis, MO, USA), Fisher Scientific (Fair Lawn, NJ) or Fluka (St. Louis, MO). Biocytin was purchased from Sigma-Aldrich. Neurons filled with biocytin were fixed (12–24 h) with paraformaldehyde (4%) after recording, then washed in 1X PBS solution. Biocytin staining was carried out with Vector PK4000 and SK4100 kits (Vector Laboratories, Burlingame, CA, USA). Tiled z-stack image acquisition was performed using a Zeiss AxioImager microscope with an AxioCam MRc camera (Zeiss) and ZEN software (blue edition; Zeiss) at 100× or 63× magnification.

Sparse labeling of cerebellar Purkinje cells was achieved by *in utero* ventricular viral injection (100 nL per ventricle) at embryonic day 14 (E14) with an adeno-associated virus (Pseudo type 2.1) carrying a GFP payload expressed under the CAG promoter. Once animals reached post-natal day 30 (P30) they were transcardially perfused with 4% paraformaldehyde fixative. Fixed brains were then sectioned at 100 μm thickness (horizontal plane) using a Leica micro-slicer and mounted for microscopy. Tiled z-stack image acquisition was performed using a Zeiss 880 confocal microscope at 63× magnification.

### System Requirements and Installation

ShuTu consists of two parts: one for processing images and automated reconstruction, and the other for viewing and editing the morphology using graphical user interface (GUI). The software requires installation of Open MPI and C compiler. The software package was tested on a desktop computer with Intel Core i7-4770 CPU@3.40GHz CPU and 16 GB memory, running Ubuntu 14.04 LTS. These are typical settings for current high-end desktop computers. Multiple processors are desirable since the algorithms are designed to utilize multiple processors to speed up computation. However, the memory usage must be monitored to make sure that the demand on memory does not exceed 100%. The number of processors used is specified as a parameter for the command line when running the code for automated reconstruction (see the section “Automated reconstruction” below).

ShuTu can be downloaded from


http://personal.psu.edu/dzj2/ShuTu/,


or


https://www.janelia.org/shutu.


An installation script is provided for Ubuntu and Mac OSX systems. This download includes all source codes for processing images and automatic reconstruction. It also includes the GUI program for viewing and editing the morphology. The source code for the GUI program is available at


  https://github.com/tingzhao/ShuTu.


On Windows 10, one can install the Ubuntu App and proceed as in Ubuntu, except that the GUI program is downloaded and installed separately and runs in Windows system.

In the directory of ShuTu, one can run


sudo sh build.sh,


which checks and installs necessary software including Open MPI. The C programs are also compiled.

### Image Acquisition and Processing

The software works with tiles of tiff stacks covering the entire neurons. Nearby tiles overlap, typically by 20%, to help fine tune the relative positions of the tiles (“stitching”). The names of the tiff stacks use the convention of a common string (filenameCommon followed by a number and .tif). With the *x, y* positions of the tiles specified, one can use the program stitchTiles to stitch the tiles. The results are stored in file filenameCommon.json.

Modern microscopes often allow automatic generations of overlapping tiles of tiff stacks. In our case, we imaged hippocampal neurons with Zeiss Axio Imager with AxioCam and Zen blue software. Once the boundary in the field of view (*XY*) and the range of the depths (*Z*) that contain the neuron are set, the images at each tile position and depths are automatically taken, and the positions of the image are stored in an xml file. The filenames of these images contain information about the tile number and depth. Using them, we assemble all images at different depths for each tile into one tiff stack. The command is


mpirun -n numProc ./createTiffStacksZeiss 
dirData filenameCommon


Here numProc is the number of processors to be used; and dirData is the path to the directory in which the xml file resides. The images of the planes are stored in a subdirectory. filenameCommon is the common part of the names given by the user to the created tiff stacks.

The user can generate the overlapping tiff stacks in other ways. The files should be named in the format of filenameCommon1.tif, filenameCommon2.tif, etc.

The tiff stacks are preprocessed using the command


mpirun -n numProc ./processImages dirData


If the images are dark-field, the command should be


mpirun -n numProc ./processImages dirData 1


In this case, the images are inverted into bright-field images. The original images are renamed by adding .org.tif to the end of the original file names, and are moved to a directory OriginalImages.

Stitching the images is done with program stitchTiles. It is assumed that in dirData there exists the xml file, generated by the Zen Blue software during automatic image acquisition; or a text file tileSequences.txt, with the following format explained with an example:


80
1, 2, 3, 4
1, 5
5, 6, 7, 8
6, 9
9


The first line is a single number specifying the shifts in percentages (100% overlap). The second line specifies the tile numbers of the first row scanning from left to right. The third line specifies two connected tiles in the first row and the second row. The fourth line specifies the tile numbers in the second row. This continues until the last row is specified. With one of these files in the directory, the command for stitching is


mpirun -n numProc ./stitchTiles dataDir


After stitching is done, one can proceed to reconstruct the neuron semi-automatically using the GUI program of ShuTu (see Appendix 1). Another choice is to run the automatic reconstruction algorithm to create a draft reconstruction and edit it using the GUI program (see below).

In stitching, the precise offsets of nearby tiles are computed by maximizing phase correlation (Zitova and Flusser, 2003). Using the maximum spanning tree algorithm (Graham and Hell, 1985), a tree graph connecting all tiles and maximizing the sum of phase correlations along the connected nearby tiles is computed and used to set the relative coordinates of all tiles.

If the entire neuron is contained in a single tiff stack, the above processing steps should be skipped.

### Automated Reconstruction

The code for automated reconstruction is parallelized with MPI protocol, and runs with the command


mpirun -n numProc ./ShuTuAutoTrace dataDir
ShuTu.Parameters.dat,


where ShuTu.Parameters.dat is a text file that contains the parameters. This creates an SWC file filenameCommon.auto.swc in dataDir, which can be loaded in ShuTu for manual editing (File→Load SWC).

To automatically reconstruct neurites in a single tiff stack, one can run


./ShuTuAutoTraceOneStack dataDir/filename.
tif ShuTu.Parameters.dat.


The resulting SWC file is stored in filename.tif.auto.swc in dataDir. This should be used if the entire neuron is stored in a single tiff stack. It is also useful for tuning the parameters. Note that if the image is dark field, one needs to edit the parameter file and set the image type parameter to 1.

## Data Availability Statement

All datasets generated for this study are included in the article/Supplementary Material, and at http://personal.psu.edu/dzj2/ShuTu/.

## Ethics Statement

The animal study was reviewed and approved by Institutional Animal Care and Use Committee of the Janelia Research Campus.

## Author Contributions

DJ and TZ devised the algorithm and software. DH and C-LH performed patch-clamp recordings and imaged neurons. RT imaged neurons. DJ and NS wrote the paper.

### Conflict of Interest

The authors declare that the research was conducted in the absence of any commercial or financial relationships that could be construed as a potential conflict of interest.
