# Supplementary material for: ShuTu: Open-Source Software for Efficient and Accurate Reconstruction of Dendritic Morphology
Source: Front Neuroinform. 2019 Oct 31;13:68. doi: 10.3389/fninf.2019.00068 (PMC6834530; doi:10.3389/fninf.2019.00068)
Supplement: Supplementary file 1 [file Data_Sheet_1.pdf]

Supplementary Material for *ShuTu: Open-Source Software for Efficient and Accurate Reconstruction of Dendritic Morphology*

Dezhe Z. Jin[1*], Ting Zhao[2], David L. Hunt[2], Rachel P. Tillage[2], Ching-Lung Hsu[2], Nelson Spruston[2*]

1 Department of Physics and Center for Neural Engineering, the Pennsylvania State University, University Park, Pennsylvania, U.S.A
2 Janelia Research Campus, Howard Hughes Medical Institute, Ashburn, Virginia, U.S.A

∗ Corresponding authors: Dezhe Z. Jin, dzj2@psu.edu, Nelson Spruston, sprustonn@janelia.hhmi.org

Abbreviated title: **S**huTu

Comparison to other algorithms for automatic reconstruction

In this section, we provide further comparisons of accuracy of ShuTu, neuTube and Vaa3D on automated reconstruction of one tile covering part of the basal dendrite (Fig. 15).

A traditional way of assessing the accuracy of automated reconstructions is to compare to manual reconstructions (Acciai et al., 2016). We therefore also manually reconstructed dendrites in the tile utilizing the manual tracing capability of ShuTu's GUI (see Fig. 11). We then compared the automated reconstructions to this manual reconstruction. Specifically, we computed the total length of the dendrites in the automated reconstruction that are within the vicinity of the manual reconstruction ("correct dendritic length"). The vicinity is defined as the volume around all pairs of connected SWC points, formed by two half cylinders and a trapezoidal prism (Fig. 6a). The edges of the prism are tangential to the spheres centered at the two SWC points, with the diameters set to those of the SWC points or at least 0.4 μm. The height is set to the larger of the two diameters or at least 3 μm. If the centers of the two connected SWC points in the automated reconstruction are within the vicinity, the distance between them are added to the correct dendritic length. Of the total dendritic length in the automated reconstructions, the percentage of the correct dendritic length was 97%, 91% and 55% for our algorithm, neuTube and Vaa3D, respectively. We also calculated the total dendritic length missed by automated reconstruction by subtracting the correct dendritic length from the total dendritic length of the

manual reconstruction. Of the total dendritic length in the manual reconstruction, the percentage of the missed dendritic length was 8%, 32% and 46% for our algorithm, neuTube and Vaa3D, respectively. Hence our automated reconstruction covers more dendrites (see Fig. 15a-c). These measures show that our algorithm produced more accurate reconstruction than neuTube or Vaa3D.

Appendix 1: Editing commands for ShuTu

Loading a project

A reconstruction project can be opened by clicking on `Open Project` icon or `File` → `Open Project`. In the directory of the neuron, there should be a file `filenameCommon.tiles.json`, which is created after stitching the tiff stacks. Clicking on it opens `Tile View`, in which the 2D projections of the tiff stacks are shown. The 2D projection of the neuron should be visible. If there is a previous reconstruction of the neuron, which is stored in a file `filenameCommon.swc`, it will be automatically loaded and overlaid onto the 2D projection. The SWC file generated by the automated algorithm, `filenameCommon.auto.swc`, can be loaded by selecting `File` →`Load SWC`.

Double clicking on any tile in the `Tile View` loads the corresponding tiff stack in `Stack View`. The loaded SWC points are overlaid onto the tiff stack. To go up and down in the z-dimension, use the right and left arrow keys. The functions of the arrow keys can be also performed with mouse wheel or track pad when available.

Clicking on `Make Projection` button creates 2D projection of the tiff stack. The user can specify the number of subdivisions used in the projection. All of the projections of the subdivisions are contained in the `Projection View`, which can be browsed with the left and right arrow keys.

The SWC structure is also displayed in `3D View`. It can be rotated with the arrow keys, and shifted with the arrow keys while pressing the `Shift` key.

In all views, zoom is controlled with + and - keys. After zooming in, different parts of the images can be navigated by pressing-dragging the mouse.

If the neuron is contained in a single tiff stack, load the tiff stack with File → Open. Other steps are the same as described above. Dark field tiff stack should be converted into bright field stack with Tools → Invert Intensity.

Editing SWC points

The SWC structure can be edited in Stack View, Projection View, and 3D View. All editing can be reversed by Ctrl-z (or Command-z in Mac). Colors of SWC points indicate their topological roles in the structure: yellow and blue indicate the end points of branches; green the branching points; and red the interior points. Lines between SWC points indicate their connectivity.

In Stack View, an SWC point is plotted with a circle at its xyz position in the tiff stack. The radius of the circle is the same as that of the SWC point. As the focus plane shifts away from the z of the SWC point, the circle shrinks with its color fading. This helps the user to visually locate the z of the SWC points and inspect whether the positions and radii of the SWC points match the underlying signals of the neurites in the tiff stack.

Extension is the most commonly used editing function. In Stack View, it can be done in two ways. The first is manual extension. Click an SWC point to extend, and the cursor becomes a circle connected to the SWC point. Focus on the target neurite using the arrow keys, and match the radius of circle with that of the neurite using e and q. Ctrl-clicking on the target points creates a new SWC point connected to the starting SWC point. (In Mac, use Command instead of Ctrl.) The second is smart extension. It is the same as manual extension, except that the user clicks without pressing Ctrl. This method allows clicking far from the starting SWC points; the algorithm fills in additional connected SWC points along the neurites with the radii and depths automatically calculated. Smart extension works well when the underlying signal is reasonably strong.

To change the properties of a particular SWC point, select it by clicking on it and pressing

`Esc` to come out of the extension mode. The radius can be changed with `e` and `q`. It can be moved with `w,s,a,d` for up, down, left, right. Pressing `x` deletes it.

To connect two SWC points, click on the first point and `Shift`-click on the second point, then press `c`. Pressing `Shift`-c after selecting two points automatically fills additional SWC points, similarly as in the smart extension. To disconnect two SWC points, select them then press `b`.

In `Projection View`, 2D projections of the subdivisions of the tiff stack are overlaid with the SWC points. In this view it is easier to spot missed branches and incorrect connections. There is also a mask-to-SWC method for tracing branches. To draw a mask along a branch, press `r`. The cursor becomes a red dot. Roughly match the radius of the dot with that of neurite with `e` and `q`. Click on the start point, then `Shift`-click on the target. A red mask will be drawn along the branch. Clicking on `Mask` → `SWC` button converts the mask into SWC points, which can be examined in detail in the `Stack View`. The mask can also be drawn manually by press-dragging the mouse along the branch. To get of out the mask drawing mode, press `Esc`.

Clicking on an SWC point selects it. Pressing `z` locates the selected point in the `Stack View`, and its z position and other properties can be further examined with the tiff stack.

The user can directly modify the connections in the `Projection View`. The operations are the same as in the `Stack View`.

In `3D View`, the user can examine and modify the connections between SWC points. Connecting or breaking connections between two SWC points is the same as in the `Stack View` and the `Projection View`. Selecting an SWC point and pressing `z` locates it in the `Stack View` for further examination and extension. This operation also loads a new tiff stack if the selected point is not in the current tiff stack.

A useful way of locating broken points in the SWC structure is the operation that selects all connected SWC points to the selected SWC point. It is done by pressing `h-3`, or right-clicking the mouse and selecting `Select` → `All connected nodes`.

After correctly connecting all SWC points belonging to the neuron, the user can delete all

noise points simply by selecting all SWC points in the neuron, right-clicking the mouse, and performing `delete unselected`.

Annotating, saving, and scaling the SWC structure

After the reconstruction is done, the user needs to annotate the SWC points as soma, axon, apical dendrite, basal dendrite. This is best done in `3D View`. In the panel `control and settings`, change `Color Mode` to `Branch Type` to reveal the types of SWC points. To annotate the soma, the user can select one point in the soma, right-click the mouse, and select `Change Property` → `Set as root`. More SWC points belong to the soma can be selected by `Shift`-clicking. Then right-click to bring up the menu, then select `Change type` and set the value to 1. The SWC points in the soma are shown in blue.

To annotate the axon, select the one SWC point closest to the soma, and press `h-1`. This selects all SWC points down stream of the selected point. Then change type to 2. Basal dendrites and apical dendrite can be similarly annotated, and their types are 3 and 4, respectively.

In the panel `control and settingings`, setting `Geometry` to `normal` produces the volume representation of the SWC structure, with surface rendered between adjacent SWC points.

To save the reconstruction, click on the objects in the panel `Objects`, which selects the corresponding SWC points. Then in the window of the SWC structure, left-click and do `save as`. It is best to use the default filename `filenameCommon.swc`.

The dimensions of the SWC points in `filenameCommon.swc` are pixel based. To convert them into physical dimensions in μm, type in the terminal

```
./scaleSWC dataDir ShuTu.Parameters.dat
```

This process uses `xyDist` and `zDist` in `ShuTu.Parameters.dat`, which specify in μm the xy pixel distance and z distance between successive planes. The results are saved in `filenameCommon.scaled.swc`.

Right after finishing the reconstruction and with `ShuTu` closed, the number of various editing operations can be analyzed using the command

```
python analyzeNEO.py
```

This requires the user to install Python 2.7. A plot similar to Fig. 13c will be generated. The script `analyzeNEO.py` parses the log file generated by `ShuTu`. The log file can contain several neuron reconstruction sessions, but the script only parses the most recent one. When estimating the total time of manual editing, idle times of the user are excluded if they are detected in the log file. The log file is assumed to be at

```
~/.neutube.z/log.txt
```

If the log file is in other locations, the user can use command

```
python analyzeNEO.py logFileDir/log.txt.
```

There are many more editing functions in ShuTu. The user can refer to `Help` for more instructions.

Shortcut keys

There are shortcut keys for many editing operations in `Stack View`, `3D View`, and `Projection View`. These are summarized in Tables 1-4.

Appendix 2: Technical details of automated reconstruction

Here we provide technical details of the automated reconstruction algorithm presented in the main text. These details should help the users to adjust parameters for their specific needs, and facilitate further development of the algorithm. The parameters in each step are summarized in series of tables. The algorithm is explained with the same example used in the main text.

Coordinate system

A tiff stack consists of successive 2D images (referred to as planes) taken at increasing depths at regular intervals. We denote a pixel in a tiff stack with coordinates (x, y, z). Here x, y are

the pixel positions in the planes, and z is the depth. We take the convention that in a plane, the x axis points vertically downwards and the y axis horizontally to the right (Fig. 1).

The distance between neighboring pixels in x and y is denoted as d_{xy}. The distance between successive planes is denoted as d_z. In the example, $d_{xy} = 0.065$ μm (`xyDist`, name in the parameter file), and $d_z = 0.5$ μm (`zDist`; Table 5).

Prepocessing

Our algorithm requires that the images are grayscale with bright background. Other image types must be converted into bright-field grayscale images, and this is done in preprocessing. In particular, color images are converted into grayscale according to

$$I(x,y,z) = 0.21 I_r(x,y,z) + 0.72 I_g(x,y,z) + 0.07 I_b(x,y,z),$$

where I is the intensity of the grayscale and I_r, I_g, I_b are those of the red, green, and blue channels. Dark-field images are inverted by subtracting the grayscale intensity at each pixel from the maximum intensity of the tiff stack. To reduce pixel noise, each plane is smoothed with 2D Gaussian filter with $\sigma = 1$ pixel. The intensity is scaled so that the maximum is 1 for the tiff stack.

2D projection

We identify neurites in a tiff stack from its minimum-intensity 2D projection. The intensity $I(x,y)$ of the 2D projection is taken as the minimum intensity among all pixels with the same z. Projections of dendritic branches form dark paths in $I(x,y)$ (Fig. 3a). Shadows of branches in out-of-focus planes (Fig. 2d) do not create separate dark paths in the 2D projection; instead, their projections flank those of the branches, forming smooth decay of intensity away from the center lines of the branches. The problem of confusing the shadows of the branches as neurites in the out-of-focus planes, as shown in Fig. 2d, does not exist in the 2D projection.

To eliminate smooth variations of the background due to uneven lighting, we subtract from

$I(x, y)$ a background, which is obtained by blurring $I(x, y)$ with a Gaussian filter with standard deviation $\sigma_b = 2$ μm (`sigmaBack`; Fig. 3b). We then normalize the range of $I(x, y)$ to $(0, 1)$ (Fig. 3c). Smaller σ_b enhances weak signals relative to strong signals (Fig. 3d). This is because the background with smaller σ_b tracks the signal strength more closely, and when subtracted, takes away more from the strong signals. But σ_b should be large enough to ensure that the subtracted background is smooth and does not weaken the signals.

Binary mask

From the 2D projection we create a binary image $b(x, y)$ to indicate pixels that belong to neurites. Specifically, $b(x, y) = 1$ for pixels in the neurites (foreground pixels) and $b(x, y) = 0$ for those in the background (background pixels). We call the area defined by the foreground pixels as binary mask.

The first step in creating the mask is convolving $I(x, y)$ with valley detectors with varying orientations, and finding the maximum and minimum responses to the detectors (Fig. 3e). A valley detector $f(x, y)$ is a patch of 2D image (or filter) consisting of an oriented dark band flanked by two bright bands. Mathematically the filter is expressed as

$$f(x, y) = \frac{1}{2\pi\sigma^2} \frac{\partial^2}{\partial\tau^2} e^{-(x^2+y^2)/2\sigma^2},$$

which is a directional second derivative of a Gaussian with standard deviation σ. Here

$$\frac{\partial}{\partial\tau} = \hat{\tau} \cdot \nabla = \tau_x \frac{\partial}{\partial x} + \tau_y \frac{\partial}{\partial y},$$

where $\hat{\tau} = \tau_x \hat{x} + \tau_y \hat{y}$ is a unit vector perpendicular to the orientation of the dark band.

Convolving $I(x, y)$ with the filter creates the response $R(x, y)$:

$$\begin{aligned} R(x, y) &= \int dx'dy' I(x + x', y + y') f(x', y') \\ &= I_{xx}\tau_x^2 + 2I_{xy}\tau_x\tau_y + I_{yy}\tau_y^2, \end{aligned} \quad (1)$$

where

$$I_{xx} = \frac{1}{2\pi\sigma^4} \int dx'dy' I(x+x', y+y') \left(\frac{x'^2}{\sigma^2} - 1 \right) e^{-(x'^2+y'^2)/2\sigma^2},$$

$$I_{xy} = \frac{1}{2\pi\sigma^4} \int dx'dy' I(x+x', y+y') \frac{x'y'}{\sigma^2} e^{-(x'^2+y'^2)/2\sigma^2},$$

$$I_{yy} = \frac{1}{2\pi\sigma^4} \int dx'dy' I(x+x', y+y') \left(\frac{y'^2}{\sigma^2} - 1 \right) e^{-(x'^2+y'^2)/2\sigma^2}.$$

We obtain the maximum or minimum response at (x, y) using the Lagrange multiplier method:

$$R' = I_{xx}\tau_x^2 + 2I_{xy}\tau_x\tau_y + I_{yy}\tau_y^2 - \lambda(\tau_x^2 + \tau_y^2 - 1).$$

At the extrema we have

$$0 = \frac{\partial R'}{\partial \tau_x} = 2(I_{xx} - \lambda)\tau_x + 2I_{xy}\tau_y,$$

$$0 = \frac{\partial R'}{\partial \tau_y} = 2I_{xy}\tau_x + 2(I_{yy} - \lambda)\tau_y.$$

These are linear equations, which can be expressed in matrix form as

$$\begin{pmatrix} I_{xx} - \lambda & I_{xy} \\ I_{xy} & I_{yy} - \lambda \end{pmatrix} \begin{pmatrix} \tau_x \\ \tau_y \end{pmatrix} = 0. \tag{2}$$

To have none-zero solutions for τ_x and τ_y, we must have

$$\begin{vmatrix} I_{xx} - \lambda & I_{xy} \\ I_{xy} & I_{yy} - \lambda \end{vmatrix} = 0,$$

where λ is the eigenvalue of the Hessian matrix. There are two solutions:

$$\lambda_1(x, y) = \frac{1}{2} \left(I_{xx} + I_{yy} + \sqrt{(I_{xx} - I_{yy})^2 + 4I_{xy}^2} \right),$$

$$\lambda_2(x, y) = \frac{1}{2} \left(I_{xx} + I_{yy} - \sqrt{(I_{xx} - I_{yy})^2 + 4I_{xy}^2} \right).$$

Here we chose $\lambda_1(x,y) > \lambda_2(x,y)$. Solving τ_x and τ_y from Eq. (2) and plugging in to Eq. (1), we find the response at the extrema:

$$R(x,y) = \tau_x^2 I_{xx} + 2 I_{xy}\tau_x\tau_y + I_{yy}\tau_x^2 = \lambda(\tau_x^2 + \tau_y^2) = \lambda.$$

Hence the maximum $R_m(x,y)$ of the responses $R(x,y)$ to valley detectors at varying orientations is given by

$$R_m(x,y) = \lambda_1(x,y).$$

To see how we can create the mask from $\lambda_1(x,y)$ and $\lambda_2(x,y)$, we exam three simple examples of synthetic 2D images containing some aspects of 2D projections of the real images containing neurites.

The first example is a Gaussian valley in y direction:

$$I(x,y) = I_0 - \frac{I_1}{\sqrt{2\pi}\sigma_s} e^{-x^2/2\sigma_s^2}.$$

Here σ_s is the scale of the widths of the valley; I_0 is the baseline intensity; and I_1 is the amplitude. This is an idealized model of the 2D projection of a dendritic segment with half-width σ_s. An ideal mask for this Gaussian valley is a rectangular strip spanning the y direction, centered long y-axis and with half-width σ_s.

$\lambda_1(x,y)$ and $\lambda_2(x,y)$ are easily calculated. We find that

$$I_{xx} = \frac{I_1}{\sqrt{2\pi}(\sigma^2+\sigma_s^2)^{3/2}} \left(1 - \frac{x^2}{\sigma^2+\sigma_s^2}\right) e^{-x^2/2(\sigma^2+\sigma_s^2)},$$

and

$$I_{xy} = I_{yy} = 0.$$

Therefore,

$$\lambda_1 = \begin{cases} I_{xx}, \text{ if } I_{xx} \geq 0, \\ 0, \text{ if } I_{xx} < 0. \end{cases}$$

$$\lambda_2 = \begin{cases} 0, \text{ if } I_{xx} \geq 0, \\ I_{xx}, \text{ if } I_{xx} < 0. \end{cases}$$

We can obtain a mask close to the ideal mask by thresholding $\lambda_1(x, y)$. If we set the foreground pixels as those with $\lambda_1(x, y) > 0$, the boundary of the mask is given by

$$x_b = \pm\sqrt{\sigma^2 + \sigma_s^2}.$$

The half-width of the mask is $\sqrt{\sigma^2 + \sigma_s^2}$, and it is larger than σ_s. Taking $\sigma \to 0$ leads to the ideal mask. For finite σ, it is possible to set a higher threshold for $\lambda_1(x, y)$ and obtain the ideal mask; but this requires a threshold that depends on the width of the valley.

From this example we see that we can obtain a mask that closely follow dendritic branches by thresholding the maximum responses to the valley detectors, $\lambda_1(x, y)$. The threshold should be larger than 0. Larger σ for the detectors tends to broaden the mask; therefore it is desirable to have small σ to obtain masks that closely cover the dendritic branches.

The second example is a Gaussian blob:

$$I(x, y) = I_0 - \frac{I_1}{2\pi\sigma_s^2} e^{-(x^2+y^2)/2\sigma_s^2}.$$

This is an idealized model for the 2D projections of spills created during the staining process (Fig. 2a). Such spills are noise that should be eliminated; therefore the ideal mask for a Gaussian blob should be empty.

We find that

$$I_{xx} = \frac{I_1}{2\pi(\sigma^2 + \sigma_s^2)} \left(1 - \frac{x^2}{\sigma^2 + \sigma_s^2}\right) e^{-(x^2+y^2)/2(\sigma^2+\sigma_s^2)},$$

$$I_{xy} = -\frac{I_1 xy}{2\pi(\sigma^2+\sigma_s^2)^2} e^{-(x^2+y^2)/2(\sigma^2+\sigma_s^2)},$$

$$I_{yy} = \frac{I_1}{2\pi(\sigma^2+\sigma_s^2)} \left(1 - \frac{y^2}{\sigma^2+\sigma_s^2}\right) e^{-(x^2+y^2)/2(\sigma^2+\sigma_s^2)}.$$

Therefore,

$$\lambda_1(x,y) = \frac{I_1}{\pi(\sigma^2+\sigma_s^2)} e^{-(x^2+y^2)/2(\sigma^2+\sigma_s^2)},$$

$$\lambda_2(x,y) = \frac{I_1}{\pi(\sigma^2+\sigma_s^2)} \left(1 - \frac{x^2+y^2}{\sigma^2+\sigma_s^2}\right) e^{-(x^2+y^2)/2(\sigma^2+\sigma_s^2)}.$$

We see that thresholding the maximum responses λ_1 creates a circular mask, which is far from the desired empty mask. To suppress creating foreground pixels for the Gaussian blob, additional criteria for the mask are needed. We notice that near the center of Gaussian blob, λ_1 and λ_2 are approximately equal. This motivates another criterion for the mask: in addition to λ_1 being greater than a threshold, the foreground pixels must satisfy the condition $\lambda_1 > \alpha_\lambda |\lambda_2|$, where $\alpha_\lambda > 1$ is a factor. This criterion should suppress foreground pixels for the Gaussian blob, except around a ring near the radius $\sqrt{\sigma^2+\sigma_s^2}$, where λ_2 is close to zero. This is not the ideal mask for Gaussian blob, but it is close. Note that this additional criterion does not affect the mask for the Gaussian valley in the first example, hence does not interfere with detection of dendritic branches.

The third example is a random image, which has a mean intensity I_0 and no correlations between the pixels:

$$< (I(x,y) - I_0)(I(x',y') - I_0) >= \sigma_I^2 \delta(x-x', y-y').$$

Here σ_I^2 is the variance of the pixel intensity. This is an idealized model for random pixel noise in the real images. The ideal mask should be empty.

It is easy to see that

$$< I_{xx} >=< I_{xy} >=< I_{yy} >= 0.$$

Additionally,

$$< I_{xx}^2 >=< I_{yy}^2 >= \frac{3\sigma_I^2}{16\pi\sigma^6},$$

$$< I_{xy}^2 >=< I_{xx}I_{yy} >= \frac{\sigma_I^2}{16\sigma^6},$$

$$< I_{xx}I_{xy} >=< I_{yy}I_{xy} >= 0.$$

From these we find the mean of the responses

$$< R(x,y) >= 0,$$

and the variance

$$\sigma_R^2 =< R(x,y)^2 >= \frac{3\sigma_I^2}{16\pi\sigma^6},$$

where we have used $\tau_x^2 + \tau_y^2 = 1$. σ_R represents the range of the responses expected from random fluctuations of the intensity. To avoid creating foreground pixels for random fluctuations, we should set the threshold for λ_1 larger than σ_R. But a threshold that is too large diminishes the mask for dendritic branches. Therefore the threshold for λ_1 must be chosen to preserve the signals while suppressing random noise. Inevitably, some foreground pixels to noise are unavoidable, which creates random speckles in the mask (Fig. 3h).

A guideline for selecting the length scale σ in the valley detectors can be devised by combining the insights from the Gaussian valley and the random image. The peak of the maximum responses from the Gaussian valley, which occurs at $x = 0$, is given by

$$\lambda_{1,\max} = \frac{I_1}{\sqrt{2\pi}(\sigma^2 + \sigma_s^2)^{3/2}}.$$

Comparing this to the variance of the responses to the random image, we can define the signal-to-noise ratio as

$$\rho_s = \frac{\lambda_{1,\max}}{\sigma_R} = \frac{4I_1}{\sqrt{6}\sigma_I}\left(\frac{\sigma^2}{\sigma^2 + \sigma_s^2}\right)^{3/2}.$$

This ratio is an increasing function of σ. Therefore, a large σ is useful for suppressing noise. However, a large σ overestimates the width of the valley (given by $\sqrt{\sigma^2 + \sigma_s^2}$), leading to widening of the foreground pixels. For real images, such widening can create a mask that merges nearby branches, leading to an inaccurate representation of the neuronal structure. Hence the choice of σ is a compromise between enhancing the signal-to-noise ratio while avoiding the merger of nearby branches in the mask. When the intensity fluctuation is small, we can select a small σ, leading to an accurate mask. If the fluctuation is large, we need to choose a large σ and live with the imperfect mask.

Based on the insights gained from the examples discussed above, we formulate the following procedure for creating the mask $b(x, y)$ from $\lambda_1(x, y)$ and $\lambda_2(x, y)$. Select σ of the valley detector such that the neurites are clearly visible in $\lambda_1(x, y)$ (Fig. 3d). Set $b(x, y) = 0$ with $\lambda_1 < \alpha_\lambda |\lambda_2|$ to suppress circular blobs in the 2D projection. Select a threshold θ_λ above the noise level, and set $b(x, y) = 1$ if $\lambda_1(x, y) > \theta_\lambda$ and $b(x, y) = 0$ otherwise (Fig. 3e). Since pixels belonging to the neurites tyically have higher λ_1 compared to those with random fluctuations, we set the threshold θ_λ such that the fraction of pixels selected to the mask is f_λ. For our example neuron, the parameter values are: $\sigma = 0.1$ μm (`sigmaFilter`), $\alpha_\lambda = 10$ (`lambdaRatioThr`), and $f_\lambda = 0.1$ (`sparse`; Table 6).

The binary mask generated as above is noisy, and the boundaries for neurites are rugged (Fig. 3h). To clean up noise and smooth the boundaries, we use the sparse-field level-set method outlined in (Lankton, 2009), which is a technical report based on (Whitaker, 1998). The details of level-set smoothing is as follows.

For the 2D projection $I(x, y)$ after background subtraction and normalization, we compute the gradient

$$g_r(x, y) = \sqrt{I_x^2 + I_y^2}.$$

We rescale the gradient so that the range is from 0 to 1. An edge indicator is defined as

$$g(x, y) = \frac{1}{1 + g_r^\beta},$$

where β is an exponential, typically smaller than 1, for compressing the gradient values. This function is minimal at edges of branches, where the gradients are larger. We seek a contour $\mathcal{C}$ such that the energy function

$$\mathcal{E} = \mu L[\mathcal{C}] + \oint_{\mathcal{C}} dl g(l)$$

is minimized, where $L[\mathcal{C}]$ is the total length of the contour and μ is weight parameter that controls the smoothness of the contour. The curve that minimize this energy function will be smooth and sit along the maximum gradient boundaries between the branches and the background.

The contour can be expressed as the zero-crossing points of a level set function $\phi(x, y)$. Inside $\mathcal{C}$, we have $\phi > 0$, and outside $\phi < 0$. Note that

$$L[\mathcal{C}] = \oint_{\mathcal{C}} dl.$$

The unit vectors normal to the contours in ϕ are given by

$$\hat{n} = -\frac{\nabla\phi}{|\nabla\phi|}.$$

Hence

$$L[\mathcal{C}] = \oint_{\mathcal{C}} dl\hat{n} \cdot \hat{n} = -\oint_{\mathcal{C}} dl\hat{n} \cdot \frac{\nabla\phi}{|\nabla\phi|} = -\int_{\mathcal{C}} dxdy\nabla \cdot \frac{\nabla\phi}{|\nabla\phi|}.$$

The last step uses the divergence theorem. Note that

$$-\int_{\mathcal{C}} dxdy\nabla \cdot \frac{\nabla\phi}{|\nabla\phi|} = -\int dxdyH(\phi)\nabla \cdot \frac{\nabla\phi}{|\nabla\phi|}.$$

Here $H(\phi)$ is the step function; it is 1 if $\phi > 0$ and 0 if $\phi < 0$. Integration by part gives

$$-\int dxdyH(\phi)\nabla \cdot \frac{\nabla\phi}{|\nabla\phi|} = \int dxdy\frac{\nabla\phi}{|\nabla\phi|} \cdot \nabla H(\phi) = \int dxdy\frac{\nabla\phi}{|\nabla\phi|} \cdot \nabla\phi\delta(\phi) = \int dxdy\delta(\phi)|\nabla\phi|.$$

The surface term is zero because H is zero at the boundary. Here $\delta(\phi)$ is the Dirac δ-function.

Therefore, we have

$$L[\mathcal{C}] = \int dxdy\delta(\phi)|\nabla\phi|.$$

Similarly, we can derive

$$\oint_{\mathcal{C}} dlg(l) = \oint_{\mathcal{C}} dl\hat{n}\cdot(g\hat{n}) = \int dxdy\delta(\phi)g(x,y)|\nabla\phi|.$$

Hence, we have

$$\mathcal{E} = \int dxdy\,(\mu + g(x,y))\,\delta(\phi(x,y))|\nabla\phi(x,y)|.$$

We use the variational method to find the ϕ that minimizes $\mathcal{E}$. Noting that

$$|\nabla(\phi+\delta\phi)| = \sqrt{|\nabla\phi|^2 + 2\nabla\phi\cdot\nabla\delta\phi} = |\nabla\phi| + \frac{\nabla\phi\cdot\nabla\delta\phi}{|\nabla\phi|},$$

we find

$$\delta|\nabla\phi| = \frac{\nabla\phi\cdot\nabla\delta\phi}{|\nabla\phi|}.$$

Applying integration by part, we have

$$\delta\mathcal{E} = \int dxdy\left[-\delta(\phi)\nabla\cdot\left((\mu+g)\frac{\nabla\phi}{|\nabla\phi|}\right)\right]\delta\phi.$$

Setting $\mathcal{E} = 0$, we find

$$-\delta(\phi)\nabla\cdot\left((\mu+g)\frac{\nabla\phi}{|\nabla\phi|}\right) = 0.$$

The surface term in the integration vanishes if we impose the Neumann boundary condition

$$\frac{\partial\phi}{\partial n} = 0.$$

At equilibrium and on $\mathcal{C}$ we have

$$-\nabla\cdot\left((\mu+g)\frac{\nabla\phi}{|\nabla\phi|}\right) = 0.$$

At other points, ϕ can be arbitrary. Minimization of

$$\delta\mathcal{E} = \int dxdy f[\phi]\delta\phi$$

can be done by solving the equation

$$\frac{\partial\phi}{\partial t} = -f[\phi].$$

This equation implies

$$\delta\phi = -dt f[\phi]$$

at each time step. Therefore

$$\delta\mathcal{E} = -\int dxdy f[\phi]^2 dt < 0,$$

leading to decreasing $\mathcal{E}$. In our case, we need to evolve

$$\frac{\partial\phi}{\partial t} = \nabla\cdot\left((\mu+g)\frac{\nabla\phi}{|\nabla\phi|}\right) = F$$

on the boundary. For ϕ at points other than the boundary, we need to change ϕ such that it remains a smooth function around the boundary and the second derivatives can be computed. We used the sparse-field implementation for solving this differential equation, which iteratively updates the sets of points near the boundary (Lankton, 2009). After $N_{levelset}$ number of iterations, we obtain a new binary mask by setting $b(x,y) = 1$ if $\phi(x,y) > 0$, and $b(x,y) = 0$ otherwise.

In practice, we observe that it is sufficient to smooth the initial mask by minimizing the length of the boundary alone. Hence we set the edge indicator

$$g(x,y) = 0.$$

This is because the boundaries of the initial mask are already near the neurite boundaries.

The parameter μ (`levelSetMu`) controls the smoothness of the boundary. Smoothing deletes

small noisy speckles. Larger μ creates smoother boundaries but can also cause small neurites to disappear. We set $\mu = 0.1$. Also important is the number of iterations $N_{levelset}$ (`levelSetIter`). It should be large enough to reduce noise and smooth the boundaries, but small enough not to loose structures due to over-smoothing. In our example we set $N_{levelset} = 500$.

As the final step, we remove connected pixels with total area smaller than A_s (`smallArea`). This removes noise and cleans up the mask (Fig. 3f). In the example we set $A_s = 1\ \mu\text{m}^2$.

Parameters for creating the mask are listed in Table 6.

Creating SWC points from the mask

Using the mask, we create the SWC points that describe the dendritic structure. The (x, y) positions of the SWC points are placed along the centerlines of the mask. The radii r are set as the shortest distances to the boundaries of the branches from the centerlines. The z positions are computed using the centerlines and the tiff stack.

The centerlines of the mask are obtained by skeletonization (Zhang and Suen, 1984). The skeleton is computed by iterative thinning of the mask based on the pixel values in the 8 neighboring points (Zhang and Suen, 1984) (Fig. 4a). The distance from a pixel in the centerline to the nearest boundary is computed using the Euclidian distance transformation (Danielsson, 1980) (Fig. 4b).

The depths z of the points on the centerlines of the mask are found in two steps. First, the centerlines are dissected into xy-paths (Fig. 4a). The xy-paths with length smaller than $l_{sm} = 0.5\ \mu\text{m}$ (`smallLen`) are considered as noise and excluded. Second, a z-image is created by following the xy-path and cutting through the tiff stack (Fig. 4c). A dark valley in the z-image spanning from the left edge to the right edge indicates the branch whose 2D projection falls on the xy-path (Fig. 4c). A line through the valley can be found by evaluating all paths from the left edge to the right edge (red dotted line in Fig. 4c). Specifically, a left-right path in the z-image starts from a point at the left edge. The next point is selected from the three nearby points to the right (the change in z is -1, 0, or 1). This process iterates until the right edge is reached. For

each left-right path, we compute the weighted distance, which is the sum of the distance between consecutive pixels multiplied by a weight $e^{\alpha_d I}$, where $\alpha_d = 20$ (`alphaDistance`) is a parameter and I is the intensity of the right point. The weight penalizes bright pixels, and encourages the left-right path to go through dark pixels. The left-right path with minimal weighted distance, or the shortest path, is selected. The path follows the dark valley spanning from the left edge to the right edge, as shown in Fig. 4c (dotted red line). The z values of this shortest path gives the depth for each point in the xy-path. A large α_d ensures that the shortest path follows dark pixels. But a value too large can lead to distortions of the path due to dark spots from other branches or noise.

Linked SWC points are placed on the xy-path. The distance between successive SWC points is set to be at least 1.2 times the radii (for points near som, 0.5 times radii). We check the validity of SWC points, remove any invalid ones. Adjacent SWC points are connected along the xy-path. Removal of invalid SWC points may have created a large distance between two consecutive SWC points; in this case, we do not connect them. The criterion is

$$d_{12} > \alpha_{xy}(r_1 + r_2),$$

where d_{12} is the Euclidian distance in xy between the two SWC points; r_1, r_2 are the radii; and $\alpha_{xy} = 2.0$ (`distFactConn`) is a factor. Sharp turns in xy-path often result from errors in the skeleton due to crossing branches. To avoid this problem, we do not connect the two SWC points if doing so creates a lage angle (greater than $\theta_{thr} = \pi/3$, `angle`) between consecutive lines connecting the SWC points. We also do not connection the SWC points if the z difference between them is too large, as this often leads to errors in connecting branches far way in z. The criterion is

$$d_z|z_1 - z_2| > \alpha_{zj} d_{xy}(r_1 + r_2),$$

where $d_z = 0.5\ \mu m$ is the distance between successive planes in the tiff stack; $d_{xy} = 0.065\ \mu m$ is the pixel distance in xy; and $\alpha_{zj} = 3.0$ (`zJumpFact`) is a factor for adjusting the threshold.

The parameters for creating SWC points are listed in Table 7.

Checking the validity of an SWC point

The SWC points created from the mask can be incorrect. For example, if some branches are parallel to each other and are very close, they can be merged in the mask, leading to incorrect SWC points. Therefore it is important to check the validity of the SWC points and reject incorrect ones. Since the mask can be imperfect, we check the validity not with the mask but with the original tiff stack. The main idea is that a valid SWC point should sit on the centerline of a valley in the plane at the depth of the SWC point.

Consider an SWC point at (x_p, y_p, z_p) with radius r_p. The validity of the point is tested with the intensity $I(x, y)$ of pixels in the plane at $z = z_p$ (Fig. 5a). We take a local square patch in the plane centered at (x_p, y_p) with size set to $\max(4r_p, 2r_{min})$, where $r_{min} = 2$ μm (`minRange`). Pixel intensity across the patch can have a tilt, such that one side is much brighter than the other. This could be due to uneven lighting, or shadows cast by nearby dark neurites. To reduce the adverse effects of tilt, we least-square fit the intensity with a linear function $a(x - x_p) + b(y - y_p) + c$, where a, b, c are the fitted parameters, and subtract the fitted function from $I(x, y)$ in the patch. The results are shifted and scaled so that the intensity range is the same as that before subtraction.

Ideally, the SWC point should be at a local center of a valley in $I(x, y)$ of a dendritic branch. To test this, we create a profile of intensity along a line through (x_p, y_p) and at angle θ relative to the x-axis (Fig. 5a), and test the existence of an inverse peak. The profile is a one-dimensional curve $I_\theta(d)$ (black line, Fig. 5b), where d is the coordinate of a pixel point on the line, with (x_p, y_p) set as the origin. $I_\theta(d)$ is the intensity value at the pixel point. The range of $|d|$ is limited to $d_{\max} = \min(2r_p, r_{min})$. We obtain a smoothed profile $I_{s,\theta}(d)$ by convolving $I_\theta(d)$ with a Gaussian filter with $\sigma_s = 0.2$ μm (`sigmaSmoothCurve`, green line, Fig. 5b), and detect the inverse peak in $I_{s,\theta}$. We take θ to be multiples of $\pi/8$. Hence there are 8 profiles (Fig. 5a,c).

We evaluate the existence of an inverse peak in the smoothed profile $I_{s,\theta}$ using two cri-

teria. The first criterion ensures that the inverse peak is deep enough. Specifically, we require that the local minimum of $I_{s,\theta}$ near (x_p, y_p) is smaller than a threshold $I_{th} = I_b - \alpha_{th}\sigma$ (gray line, Fig. 5b). Here I_b is the baseline, and is set to 80 percentile value of the intensity in the patch; $\sigma = 0.03$ (`sigma`) is the estimate of the fluctuation level of the intensity; and $\alpha_{th} = 1$ (`factSigmaThreshold`) is an adjustable factor. This factor is increased to $\alpha_{th} = 2$ (`factSigmaThreStrict`) during creation of SWC points from the mask to make the validity judgement stricter, which reduces noise in the SWC structure. The second criterion ensures that the inverse peak has two flanks. This is done by checking that $I_{s,\theta}$ rises above a threshold set at the mean of the maximum and the minimum of $I_{s,\theta}$ at either side of the local minimum point (gray dashed line, Fig. 5b).

If the peak is deep enough and there are two flanks, we determine the width of the peak starting from the minimum of $I_{s,\theta}$. We take a derivative of $I_{s,\theta}$ and smooth it to get dI_s (Fig. 5e). We determine the maximum absolute value of these derivatives $dI_{\max}$. Starting from the minimum point, we trace the negative part of the derivative. The tracing stops once dI_s starts to increase and $-dI_s > 0.5dI_{\max}$. This way of stopping ensures that the tracing picks out the first significantly large absolute value in the derivative and does not stop because of small bumps in $dI_{s,\theta}$. Similarly, we trace the positive part of the derivative, and stops when dI_s starts to decrease and $dI_s > 0.5dI_{\max}$. If either of these tracing does not stop before reaching the end, the peak is invalid. Otherwise, the width w of the peak is set to the distance between the two stopping points (black vertical lines, Fig. 5e). The derivatives of all eight smoothed profiles are shown in Fig. 5f. To ensure that fluctuations between the two stopping points are small enough, we check the peaks in $|dI_s|$. If the maximum of these peaks is larger than $0.5dI_{\max}$, the profile is judged to have no valid peak.

If none of the profiles have valid inverse peaks, the SWC point is invalid. Otherwise, we choose the profile with the minimum width among the valid ones, and assign its peak position as the position (x_m, y_m) of the SWC point, and set $r_m = \alpha_r w/2$, where the factor $\alpha_r = 1.0$ (`factAdjustRadius`) is an adjustable factor introduced to enable the user to adjust the radii

of SWC points according to subjective judgement. If the distance between the original position (x_p, y_p) and (x_m, y_m) exceeds $\alpha_{shift} r_m$, where $\alpha_{shift} = 2$ (`factShift`), then the SWC point has shifted too much, and it is flagged as invalid. If r_m is smaller than $r_{min} = 0.3$ μm (`minRadius`) or larger than $r_{max} = 10$ μm (`maxRadius`), the radius of the SWC point is too small or too large, and the point is invalid.

Finally, we check whether the pixels within a radius $0.5r_m$ from the center are dark enough. Specifically, we check whether the maximum value of the smoothed profile in the orthogonal direction (red line, Fig. 5d) to the chosen profile (cyan line,Fig. 5d) within $0.5r_m$ are smaller than a threshold (green horizontal line, Fig. 5d), which is set as the maximum smoothed intensity of the chosen profile at r_m plus σ. If not, the SWC point in invalid. This check ensures that the SWC points created along edges of thick dendrites or the soma are eliminated.

If the SWC point passes all the tests described above, it is judged as valid, and (x_m, y_m, z) and r_m are set as the new position and radius. The position and radius of the SWC are thus corrected after the validity check. To ensure that the corrections converge, the checking procedure is iterated three times with the positions and radius updated. The SWC point is accepted if it passes the tests all three times. The angle θ of the profile denotes the orientation perpendicular to the branch (red line, Fig. 5a).

The parameters used in checking the validity of an SWC point are listed in Table 8.

Adjusting z

Checking validity of an SWC point shifts its xy position. The shift can be significant, especially during the creation of SWC points using the mask, since the mask can deviate from the underlying neurites significantly if there are close branches. To ensure that z of the SWC point is accurate, we further adjust z. We create the intensity profile along z at the xy position (black line, Fig. 4c), then smooth it with parameter $\sigma_{s,z} = 2$ (`sigmaSmoothCurveZ`; green line, Fig. 4c).

We check the existence of inverse peak in the smoothed profile, similarly as done for the intensity profiles used for checking validity of SWC points. We estimate the fluctuations in

the profile by computing the standard deviation σ_z of the difference between the original and the smoothed profiles. We smooth the derivative of the smoothed profile, and determine the maximum $d_{z,max}$ of the absolute values of the derivatives. If $d_{z,max} < \alpha_z \sigma_z$, it is judged that there is no inverse peak, and the SWC is judged as invalid. Here $\alpha_z = 0.1$ (`factSmallDerivZ`) is a factor. Otherwise, the extent of the inverse peak centered around the original z position is decided by finding the first places away from the center at which the smoothed profile stops increasing. A threshold is set to the maximum value at these two ends minus $\alpha_{th,z}\sigma_z$. Here $\alpha_{th,z} = 1$ (`factSigmaThresholdZ`) is a factor. If the minimum value within the extent is smaller than this threshold, the inverse peak is valid, and the z is adjusted to the position of the minimum. Otherwise the SWC is judged invalid.

The parameters for adjusting z are listed in table 9.

Mark pixels occupied

To avoid creating duplicated SWC points, we mark pixels in the tiff stack in the vicinity of the existing SWC points as occupied. Before creating a new SWC point, we check whether the pixel at its center is marked as occupied; if so, the SWC point is not created.

The marked pixels around two connected SWC points (x_1, y_1, z_1, r_1) and (x_2, y_2, z_2, r_2) are in a volume formed by two half cylinders with radii $\alpha_{occ} r_1$ and $\alpha_{occ} r_2$, respectively, and a trapezoidal prism that fits with the half cylinders (Fig. 6a). Here $\alpha_{occ} = 1$ (`factMarkOccXY`) is a parameter for adjusting the extent of exclusion in xy. The z extent of the marked volume is large enough to contain the two SWC points: the distances from the top and bottom planes of the volume to the nearest SWC points are set to the maximum of $\alpha_{occ} r_1$, $\alpha_{occ} r_2$, or $z_{occ} = 3$ μm (`zOcc`). Increasing the volume of the marked pixels prevents creations of spurious SWC points. However, if the volume is too large, correct SWC points can be eliminated, especially when branches are close to each other. These opposing constraints should guide the choice of the appropriate size for the volume.

The parameters for marking pixels occupied are in Table 10.

Thick dendrites and the soma

Thick dendrites and the soma can be missing from the mask created with the valley detectors. There are two main reasons: (1) their dimensions are much larger than the length scale of the valley detectors; (2) the intensities of the pixels inside them are uniform. Consequently, only the pixels at their boundaries show up in the mask. This leads to the error of no SWC points for these structures. To correct this problem, we check the existence of thick dendrite and the soma in each tiff stack. The check is based on the observation that these structures are typically well stained and the pixels in them are very dark.

Specifically, we create and compare two 2D projections of the tiff stack. In the first one, we only project the pixels that are marked occupied because they are in the vicinity of the existing SWC points. We enlarge the marked volume by increasing α_{occ} and z_{occ} to three times of the original values. This is to ensure that all pixels associated with the dendrites already covered the SWC points, including the shadows the dendrites in the out-of-focus planes, and completely marked. From this 2D projection we determine a threshold, which is set to the intensity of the darkest 5% of pixels. This threshold indicates the darkness of the pixels covered by the existing SWC points.

In the second 2D projection, we project only the pixels that are not marked occupied. We then count the number of pixels that are darker than the threshold determined from the first 2D projection. If this number is larger than the number of pixels in an area $0.5L_{\text{soma}}^2$, where $L_{\text{soma}} = 2\ \mu m$ (`somaLengthScale`) is the length scale of the soma, we decide that the tiff stack contains thick dendrite and/or the soma since there are significant number of dark pixels that are uncovered by the SWC points.

To place SWC points on thick dendrites and the soma, we create a 2D projection of the tiff stack with all pixels, smooth it, and create a mask by selecting top $\theta_{soma} = 0.08$ (`somaSparseThr`) fraction of the darkest pixels. From the mask we create SWC points as described before. SWC points are created only if their positions are not marked occupied by the existing SWC points, using the original values of α_{occ} and z_{occ}.

The parameters for creating SWC points for thick dendrites and soma are listed in Table 11.

Extending SWC points in 3D

The SWC structures created from the masks are often incomplete, mostly due to the limitations of the masks in separating nearby branches in 2D projections. These branches could be well separated in the tiff stack although their 2D projections are not; therefore it is useful to extend the SWC structure using the tiff stack.

To minimize the interference from noise, we delete isolated SWC points that are not connected to any other SWC points. To ensure that the extension does not create duplicated SWC points, we mark pixels nearby the existing SWC points occupied (red circles, Fig. 6b). From an end SWC point (x, y, z, r) (yellow circle, Fig. 6b), we search the plane at z for the candidate for the next point. To reduce noisy fluctuations, the pixel intensity in the plane is smoothed with a Gaussian filter with $\sigma = 2$ pixels.

The search is done by finding a path starting from the end point and through the neurite (white line, Fig. 6b). To do so, we draw an arc (blue arc, Fig. 6b) of radius $r_{search} = 3$ μm (`searchMax`), or $(\alpha_{s,m} + 2)r$, whichever is greater. Here $\alpha_{s,m} = 1.2$ (`factSearchMin`) is a factor. The arc is restricted to a range of angle ($\theta_{\mathrm{thr}} = \pi/3$, `angle`), where the angle is between the line from a pixel in the arc to the end point (black lines, Fig. 6b) and the line from the end point to its connected SWC point (yellow line, Fig. 6b). From each point on the arc, we compute the intensity weighted shortest distance path to the end point (black line, Fig. 6c). The resulting profile of the distance is Gaussian smoothed with parameter 2.0 (green line, Fig. 6c). The standard deviation of the difference between the original and the smoothed profiles is taken as the reference for the fluctuations in the distances. We then search for the significant local minima in the smoothed profile. To be significant, the local minimum must be smaller than a threshold set to the mean of the maximum and the minimum of the smoothed profile, minus α_{DD} times the standard deviation. Here $\alpha_{DD} = 20$ (`factSigmaDD`) is a factor.

The shortest paths from the local minima to the end point are used to place the next SWC

point. When there are multiple paths, each path is tested sequentially. The depth of the neurite along a chosen path is computed using the xy-path technique. Starting from the end point and following the path, we test placing a new SWC point x_c, y_c, z_c, r_c. We test the validity of the candidate SWC point, which also adjusts x_c, y_c and r_c. The validity test is done three times. If the distance from (x_c, y_c) to (x, y) is smaller than $\alpha_{s,m} r$, it is not accepted. If the candidate point passes all three tests, we check if it is near an existing SWC point; if so, we check whether the existing SWC point is connectable to the end point. If connectable, the existing SWC point is added to the candidate pool for connecting the end point to the existing SWC points. If a valid candidate point does not come close to the existing SWC points, and it is connectable to the end point, we create a new SWC point at (x_c, y_c, z_c) with radius r_c. It is connected to the end point, and serves as a new end point from which the extension continues. If no new SWC point is created after checking all paths, we check the list of candidates for connections, and select the one with the minimum distance and connect it to the end point.

The parameters used in extending SWC points are listed in Table 12.

Connecting broken segments

After extending SWC points in 3D, a continuous branch can still be represented with broken segments of SWC points, especially if the underlying signal is weak or there are closely crossing branches (Fig. 2b,c). We connect these segments with heuristic rules to recover the branch continuity. To do so, we compute the Euclidean distance between all pairs of end points that are not connected and the differences in z are within the allowed range as described before. If the distance of the pair in xy is smaller than $1.5(r_1 + r_2)$, where r_1, r_2 are the radii, they are judged to be close to each other and are connected.

After connecting the nearby pairs, we consider more distant ones. If the two end points are within $\alpha_{xy}(r_1 + r_2)$ in xy, where $\alpha_{xy} = 2$ (`distFactConn`) is a factor; and if the angles between the two lines, linking the end points to their respective connected SWC points, is smaller than $\theta_{thr} = \pi/3$ (`angle`); then the two points are preserved in the candidate pool

for potential connections. We then iteratively connect the pairs of end points in the pool, connecting the closest available pairs first. Once connected, the end points are excluded from further connections. This pairwise connection stops if the pairs are all considered or if further connections create loops in the SWC structure.

The parameters used in connecting end points are listed in Table 13.

Subdividing tiff stack in z

The 2D projection can be complicated when there are many branches in one stack. This often leads to occlusions in the 2D projection and missed branches in the reconstruction. One way of mitigating this problem is to divide the tiff stack in z into $n_{div} = 8$ (`nSplit`) slabs with equal heights in z. We create SWC points separately for each slab. When creating the 2D projection for a slab, we include extra volume in z by extending the height in both directions by $z_{ext} = 3$ μm (`zext`). This is useful for getting good projections of branches that are near the dividing planes between the slabs. The z-image also includes the extended volume. Any SWC points whose depths are beyond the slab boundary are deleted. The extension from the end points are done with the entire tiff stack, which helps to connect SWC points that belong to the same branch but are cut by the subdivision.

The parameters of subdivision are listed in Table 14.

Combining SWCs for the entire neuron

The image of an entire neuron consists of multiple tiff stacks stitched together (Fig. 8a). The coordinates of the stacks relative to the first stack are determined during the stitching. For each stack we obtain the SWC structure, and shift the positions of the SWC points by the relative coordinates of the stack. The SWC points of individual stacks are read-in sequentially. To avoid duplicated SWC points in the overlapping regions of adjacent stacks, pixels near the SWC points that are already created are marked occupied by setting the parameters z_{occ} 5 times of the usual value and r_{occ} 2 times. If the position of SWC points are at the marked pixels, they are deleted.

For individual stacks, the step of connecting the end points is omitted. Instead, after reading in the SWC points of all stacks, we extend the SWC points from the end points, and then connect the new end points. To eliminate noise, we delete very short leaf branches in the SWC structure (those that have fewer than $n_{dmin} = 5$ SWC points, `minNumPointsBr`). In addition, we eliminate isolated branches that are shorter than $l_{min,iso} = 20$ μm (`minLenBrIso`). Increasing this number reduces noise in the reconstruction, but can also delete some of the correct reconstruction. The reconstructed SWC structure for the example neuron is shown in Fig. 8b-d.

The parameters are listed in Table 15.

References

Acciai L, Soda P, Iannello G (2016) Automated neuron tracing methods: an updated account. *Neuroinformatics* 14:353–367.

Danielsson PE (1980) Euclidean distance mapping. *Computer Graphics and image processing* 14:227–248.

Lankton S (2009) Sparse Field Methods - Technical Report. *Georgia Institute of Technology Techniacal Report* .

Whitaker RT (1998) A Level-Set Approach to 3D Reconstruction from Range Data. *International Journal of Computer Vision* 29:203–231.

Zhang TY, Suen CY (1984) A fast parallel algorithm for thinning digital patterns. *Communications of the ACM* 27:239–242.

Tables

<table>
<tr><th>Shortcut</th><th colspan="2">Function</th><th>Note</th></tr>
<tr><td>Ctrl/Cmd+A</td><td colspan="2">Select all SWC points</td><td></td></tr>
<tr><td rowspan="8">h</td><td colspan="2">Turn on selection mode (show hints too) then:</td><td rowspan="8">Hold Shift to trigger
a selection directly.
Example: Shift+1 selects
downstream points directly.</td></tr>
<tr><td>1</td><td>select downstream points</td></tr>
<tr><td>2</td><td>select upstream points</td></tr>
<tr><td>3</td><td>select neighboring points</td></tr>
<tr><td>4</td><td>select passing branches</td></tr>
<tr><td>5</td><td>select connected points</td></tr>
<tr><td>6</td><td>inverse selection</td></tr>
<tr><td>7</td><td>select small trees</td></tr>
<tr><td rowspan="4">Shift+r</td><td colspan="2">Draw selection rectangle holding the mouse then:</td><td rowspan="4">For 3D View only.
Hold Shift
to append selection.</td></tr>
<tr><td>s</td><td>select points in the rectangle</td></tr>
<tr><td>t</td><td>select trees in the rectangle</td></tr>
<tr><td>Ctrl/Cmd+t</td><td>select terminal branches
in the rectangle</td></tr>
</table>

Table 1: Shortcut keys for selecting SWC points. `Ctrl/Cmd` means substituting `Ctrl` for `Command` in Mac.

`Backspace/Delete/x`	Delete selected objects	
`c`	Connect selected SWC points	
`n`	Connect to the nearest SWC points (only works for a single selected point)	
`b`	Break SWC connections	
`i`	Insert between selected points	
`q/<`	Decrease radius	
`e/>`	Increase radius	
`Ctrl/Cmd+g`	Turn on adding new SWC point	
`Space`	Extend from selected point	
`R`	Turn on painting mask	For 2D projection only
`Ctrl/Cmd+e`	Turn on erasing mask	
`a`	Move selected SWC points to left.	For 2D projection only. Hold `Shift` for fast movement.
`d`	Move selected SWC points to right	
`w`	Move selected SWC points up	
`s`	Move selected SWC points down	
`Ctrl/Cmd+z`	Undo	
`Ctrl/Cmd+Shift+z`	Redo	

Table 2: Shortcut keys for editing SWC structure. `/` denotes equivalent keys.

`z`	Locate selected SWC points in Stack View
`=`	Zoom in
`-`	Zoom out
`Arrows`	Rotate
`Shift Arrows`	Move
`Ctrl/Cmd+s`	Save SWC structure to file
`Ctrl/Cmd+Plus`	Increase SWC size scale
`Ctrl/Cmd+Minus`	Decrease SWC size scale
`Ctrl/Cmd+g`	Change SWC display mode
`Ctrl/Cmd+b`	Toggle the mode of displaying neurons in the tile box only

Table 3: Shortcut keys for visualization of SWC structure in 3D View.

`=`	Zoom in	
`-`	Zoom out	
`a`	Move image to left.	Hold `Shift` for fast movement
`d`	Move image to right	
`w`	Move image up	
`s`	Move image down	
`Left_Arrow/e`	Decrease z position of slab	
`Right_Arrow/q`	Increase z position of slab	

Table 4: Shortcut keys for 2D Projection View.

Parameter	Name	Value	Meaning
d_{xy}	`xyDist`	0.065 μm	pixel distance in xy
d_z	`zDist`	0.5 μm	z-distance between successive planes
σ_b	`sigmaBack`	2 μm	length scale for smooth background

Table 5: Parameters for tiff stacks and 2D projections. Names of the parameters appear in the file containing parameters for automated reconstruction.

Parameter	Name	Value	Meaning
σ	`sigmaFilter`	0.1 μm	length scale of valley detector
α_λ	`lambdaRatioThr`	10	factor for eliminating circular blobs
f_λ	`sparse`	0.1	fraction for determining the threshold
μ	`levelSetMu`	0.1	parameter for level set smoothing
$N_{levelset}$	`levelSetIter`	500	number of level set iterations
A_s	`smallArea`	1.0 μm^2	threshold for small areas removed

Table 6: Parameters for creating the binary mask.

Parameter	Name	Value	Meaning
l_{sm}	`smallLen`	0.5 μm	smallest length of xy-path used
α_d	`alphaDistance`	20	factor for weighting distance between pixels
α_{xy}	`distFactConn`	2	factor for disconnecting two consecutive SWC points
θ_{thr}	`angle`	$\pi/3$	maximum angle allowed between consecutive lines
α_{zj}	`zJumpFact`	3	factor for z jump threshold

Table 7: Parameters for creating SWC points from 2D mask.

Parameter	Name	Value	Meaning
r_{min}	`minRange`	2 μm	lower bound for the range of the profiles
σ_s	`sigmaSmoothCurve`	0.2 μm	length scale for smoothing profiles
σ	`simga`	0.03	estimate of fluctuations in the intensity
α_{th}	`factSigmaThreshold`	1	factor for determining the threshold for peak
$\alpha_{th,s}$	`factSigmaThresholdStrict`	2	strict factor for the threshold for peak
α_{shift}	`factShift`	2	factor for allowing shifts in the position
r_{min}	`minRadius`	0.2 μm	lower bound for the radius
r_{max}	`maxRadius`	10 μm	upper bound for the radius
α_r	`factAdjustRadius`	1.0	factor for adjusting the radius

Table 8: Parameters for checking validity of an SWC point.

Parameter	Name	Value	Meaning
$\sigma_{s,z}$	`sigmaSmoothCurveZ`	2	parameter for smoothing z profile
α_z	`factSmallDerivZ`	0.1	factor for determining the significance of derivatives
$\alpha_{th,z}$	`factSigmaThresholdZ`	1	factor for determining the threshold for inverse peak

Table 9: Parameters for adjusting z of an SWC point.

Parameter	Name	Value	Meaning
α_{occ}	`factMarkOccXY`	1	factor for adjusting radius of the marked volume
z_{occ}	`zOcc`	3 μm	lower bound for extend of the volume in z

Table 10: Parameters for marking pixels near the SWC points occupied.

Parameter	Name	Value	Meaning
L_{soma}	`somaLengthScale`	2 μm	length scale of the soma
θ_{soma}	`somaSparseThr`	0.05	fraction for the threshold of creating mask

Table 11: Parameters for creating SWC points for thick dendrites and the soma.

Parameter	Name	Value	Meaning
r_{search}	`searchMax`	3 μm	radius for searching the next point
$\alpha_{s,m}$	`factSearchMin`	1.2	factor for the minimum distance to the end point

Table 12: Parameters for extending SWC points in 3D.

Parameter	Name Value	Meaning	
α_{xy}	`distFactConn`	2	factor for judging the closeness of end points

Table 13: Parameters for connecting end points.

Parameter	Name	Value	Meaning
n_{div}	`nSplit`	8	number of subdivisions
z_{ext}	`zext`	3 μm	extension of sub-slabs when creating SWC points

Table 14: Parameters for subdividing a tiff stack.

Parameter	Name	Value	Meaning
n_{dmin}	`minNumPointsBr`	5	minimum number of SWC points allowed in leafs
$l_{min,iso}$	`minLenBrIso`	20 μm	minimum length allowed in isolated branches

Table 15: Parameters for reducing noise in SWC structure.
